# Phytochemicals: Potential Lead Molecules for MDR Reversal

**DOI:** 10.3389/fphar.2020.00832

**Published:** 2020-06-19

**Authors:** Boshra Tinoush, Iman Shirdel, Michael Wink

**Affiliations:** ^1^Institute of Pharmacy and Molecular Biotechnology, Heidelberg University, Heidelberg, Germany; ^2^Marine Sciences Faculty, Tarbiat Modares University, Noor, Iran

**Keywords:** cancer, ABC-transporter, drug efflux, multidrug resistance, secondary metabolites, synergism

## Abstract

Multidrug resistance (MDR) is one of the main impediments in the treatment of cancers. MDR cancer cells are resistant to multiple anticancer drugs. One of the major mechanisms of MDR is the efflux of anticancer drugs by ABC transporters. Increased activity and overexpression of these transporters are important causes of drug efflux and, therefore, resistance to cancer chemotherapy. Overcoming MDR is a fundamental prerequisite for developing an efficient treatment of cancer. To date, various types of ABC transporter inhibitors have been employed but no effective anticancer drug is available at present, which can completely overcome MDR. Phytochemicals can reverse MDR in cancer cells via affecting the expression or activity of ABC transporters, and also through exerting synergistic interactions with anticancer drugs by addressing additional molecular targets. We have listed numerous phytochemicals which can affect the expression and activity of ABC transporters in MDR cancer cell lines. Phytochemicals in the groups of flavonoids, alkaloids, terpenes, carotenoids, stilbenoids, lignans, polyketides, and curcuminoids have been examined for MDR-reversing activity. The use of MDR-reversing phytochemicals with low toxicity to human in combination with effective anticancer agents may result in successful treatment of chemotherapy-resistant cancer. In this review, we summarize and discuss published evidence for natural products with MDR modulation abilities.

## Introduction

Besides surgery and radiation, chemotherapy is one of the standard treatments of cancer. Drugs used in chemotherapy usually disturb cell division by inhibition of microtubule formation or disassembly (vinca alkaloids, paclitaxel), DNA topoisomerase (camptothecin and derivatives) or they intercalate or alkylate DNA (doxorubicin, cisplatin) (Wink, 2007; Wink et al., 2012). Furthermore, chemotherapy often causes extreme side effects as these drugs also affect the division of normal cells or they cause mutations, which can lead to secondary cancers. This additionally leads to restriction of the therapeutic applications; both dosage and application interval must be kept limited. When chemotherapeutic agents are used, it is often a matter of time before the cancer cells develop resistance against them. One of the major resistance mechanisms is the overexpression of ABC transporters, which can pump out the chemotherapeutic from cancer cells. Because these ABC transporters have a wide substrate spectrum, they not only confer resistance to a single drug but to several others, therefore, the term Multidrug Resistance (MDR). Since multiple drug resistance is a major issue in tumor therapy, new strategies are necessary to overcome this obstacle. One strategy involves the combination of anti-cancer drugs with modulators of ABC transporters. Our review presents a summary about various modulating effects of phytochemicals.

### Multidrug Resistance in Cancer

One of the major difficulties in suppressing growth and survival of cancer cells is multidrug resistance. MDR is the resistance of cancer cells to various types of anticancer drugs which may have an intrinsic or acquired origin. Acquired resistance is induced after the administration of chemotherapy, whereas, intrinsic resistance already exists prior to drug application in cancer cells (Wang et al., 2019). Several mechanisms in cancer cells can lead to MDR (Coley, 2008). These include changes in target enzymes, such as DNA topoisomerases (Brown et al., 1995), alteration in microtubule-associated proteins (Zhang et al., 1998), mutations or changes in tubulin (Kamath et al., 2005; Wang and Cabral, 2005), alteration in microtubules (Kavallaris et al., 2001), mitotic arrest (Kamath et al., 2005), mutated protein p53 (O'Connor et al., 1997), disruption in DNA repair due to the damaging effect of an anticancer drug (Bernstein et al., 2002) and the impairment of apoptosis or genes involved in apoptosis and necrosis (Tanaka et al., 2000; Simstein et al., 2003).

A widespread mechanism of MDR is drug efflux via transmembrane transporters known as ATP-binding cassette transporters (ABC transporter). Overexpression of these transporters is the most important cause of drug resistance in many cancer cells. The family of ABC transporter proteins has 48 members in humans and far more in nature (Chen et al., 2016). The most well-known and widely-studied ABC transporters include P-Glycoprotein (P-gp), multidrug resistance protein 1 (MRP1), and breast cancer resistance protein (BCRP). These transporters are expressed in healthy cells of various mammalian tissues having physiological tasks for translocating small molecules. They are found especially in intestinal epithelial cells, endothelial cells of blood capillaries and epithelia of renal proximal tubules being involved in the excretion and clearance of endogenous and exogenous cytotoxic substances (Borst et al., 2000; Durmus et al., 2015; Chen et al., 2016). ABC transporters evolved in nature millions of years ago to eliminate toxic phytochemicals that herbivores would obtain from their plant diet (Wink, 2007). Anticancer drugs also can be substrates of these transporters and if being considered as foreign they get exported to the extracellular space by cells expressing ABC transporters. Cancer cells which are not resistant to anticancer agents yet can develop the ability by overexpressing these transporters for saving themselves from the substances being toxic to them. This leads to an increased efflux, leading to low intracellular drug concentrations, insufficient to kill a cancer cell. Once this overexpression has occurred, the efflux also affects other chemotherapeutics and thus, makes the cancer cell resistant to chemotherapy (Coley, 2008; Durmus et al., 2015).

### P-gp (ABCB1)

P-gp is a 170 kDa protein which is encoded by the *MDR1* gene. This transporter is found in normal cells of various tissues including the brain, liver, kidney, gastrointestinal tract and pancreas. P-gp transports anticancer drugs such as paclitaxel, doxorubicin, daunorubicin, epirubicin, mitoxantrone, vincristine, and vinblastine against the concentration gradient using energy derived from hydrolysis of ATP (Chen et al., 2016). Chemotherapeutic agents can stimulate P-gp expression in cancer cells and thereby cause resistance to chemotherapy. Chemotherapy has been reported to increase the proportion of P-gp-expressing tumors by approximately 1.8-fold in breast cancer. Moreover, in patients with activated P-gp transporter in their tumors, the risk of failure of chemotherapy is 3 times higher than in patients who do not express P-gp transporter (Trock et al., 1997).

### Multidrug Resistance Proteins (MRPs)

Another class of membrane transporters which causes MDR is MRPs. Nine members of this class have been identified so far (König et al., 2005; Coley, 2008). MRPs are found in normal cells of some mammalian tissues and expel drugs as a complex with glutathione, glucuronate, or sulfate (Borst et al., 2000; Coley, 2008). Among the MRP transporters, MRP1 (ABCC1) is the most important and most studied one regarding MDR. The MRP1 protein has a molecular weight of 190 kDa. Similar to P-gp, MRP1 expression has been reported to be considerably higher expressed in cancer cells after chemotherapy than before chemotherapy (Trock et al., 1997). Therefore, MRP1 enhances resistance to chemotherapy and to anticancer drugs such as doxorubicin, daunorubicin, epirubicin, vincristine, and vinblastine (Coley, 2008).

### BCRP (ABCG2)

Breast cancer resistance protein, also called mitoxantrone transporter (MXR1), has a molecular weight of 72 kDa. BCRP is extensively expressed in MCF-7 breast cancer cells (Doyle et al., 1998). This protein is also expressed in other tissues including the liver, kidney, and intestine (Chen et al., 2016). The anticancer drugs doxorubicin, daunorubicin, epirubicin and mitoxantrone have been described as substrates of BCRP transporter (Coley, 2008). Thus, cancer cells overexpressing BCRP transporter become resistant to these drugs.

### MDR Modulators

One of the essential requirements for developing better anti-cancer therapies is overcoming multidrug resistance. Much research has been carried out on cancer treatment and development of anticancer drugs in recent years but MDR to cytostatics is still a great impediment. Although our knowledge about the mechanisms of multidrug resistance has increased, there is no effective drug which can completely overcome or reverse resistance at non-toxic concentrations. Since ABC transporters play a fundamental role in resistance to chemotherapy, the ability to inhibit them in a combination with conventional treatments will greatly help to treat cancer (Chen et al., 2016).

Until now, different types of ABC transporter inhibitors have been examined. The use of the first generation of these compounds, including verapamil and cyclosporine A, in combination with anticancer drugs had poor clinical success and toxic effects (Daenen et al., 2004). Second generation of MDR modulators included dexverapamil, valspodar, and dexniguldipine. Even though less toxic and with a higher therapeutic index than the first generation, this group of modulators is not well suited for a therapy either, both because of its interactions with other drugs and ABC transporters, as well as due to the inhibition of enzymes like CYP3A (Wandel et al., 1999; Syed and Coumar, 2016). The third-generation ABC transporter modulators do not have the disadvantages of the first and second generation. They are potent and non-competitive inhibitors of P-gp, and also less toxic. Tariquidar (XR9576) and zosuquidar are members of the third generation of MDR modulators but unfortunately they were not efficative in clinical trials (Cripe et al., 2010; Kelly et al., 2011).

### Phytochemicals

**Figure 1:**
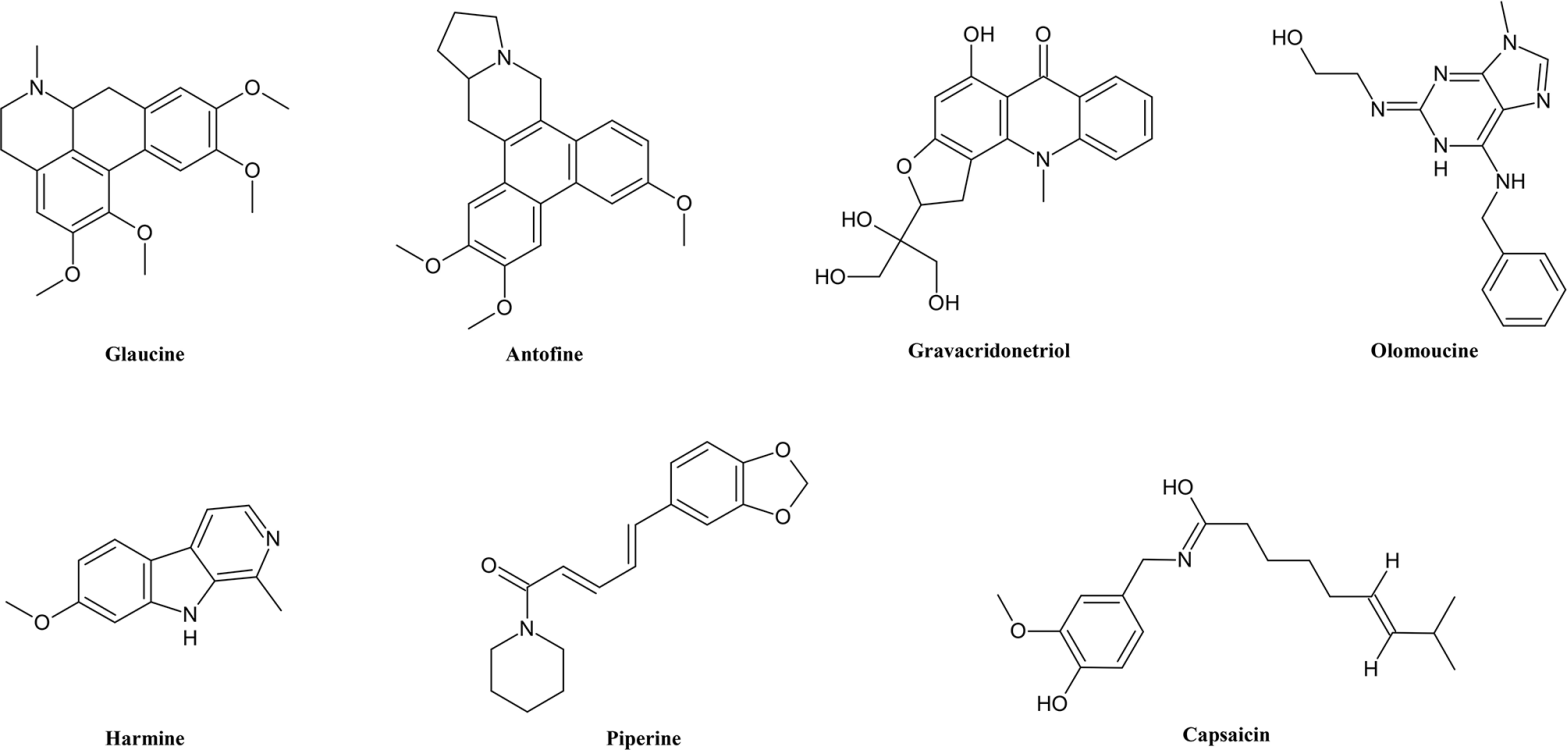
Chemical structures of some selected alkaloids with MDR reversal effects.

Alkaloids (**Figure 1**) are the most widely studied group of secondary metabolites in terms of MDR, not only because of their quantity but also because of their great diversity (Wink, 2007; Wink et al., 2012). As alkaloids have a wide distribution among angiosperms (Wink, 2020) and represent a diversity of structures, they differ in pharmacological and toxicological properties. Alkaloids contain heterocyclic nitrogen, which mostly has its origin in amino acids (Cseke et al., 2006). Alkaloids are subdivided into many subcategories of special functional groups, similarities of skeleton or biosynthetic pathways.

Quinoline and isoquinoline alkaloids for example have a benzopyridine ring differing in the position of their nitrogen. Quinazoline alkaloids have a similar aromatic structure but with two nitrogen atoms instead of one. Each of these structures has found several uses depending on molecular structure. There are many examples for quinoline alkaloids such as mefloquine as antimalarial agent, fluoroquinolone antibiotics and topotecan as anticancer drug, just to name a few (Collin and Höke, 2000; Tiwary et al., 2015). The latter two work as inhibitors of different DNA topoisomerases (Lemmer and Brune, 2004).

Quinolizidines also are cyclic nitrogen-containing compounds but unlike the previously mentioned subgroups they are not aromatic. A natural representative is sparteine, which is used as an antiarrhythmic agent blocking sodium channels (Ruenitz and Mokler, 1977; Körper et al., 1998; Gawali et al., 2017).

Among other groups of alkaloids, indole, monoterpene indole and β-carboline alkaloids show many pharmacological activities (Gilbert, 2001). A typical basic structure of β-carboline alkaloids consists of benzene fused with a five-membered pyrrole and is consequently similarly structured to some endogenous hormones and neurotransmitters such as serotonin and melatonin. In addition to benzene, the pyrrole ring of β-carboline is fused to a pyridine, another six-membered nitrogen-containing ring. This structure by itself is an inverse agonist of GABA-receptors, which involves psychological influence on humans (Aktories et al., 2017). Substrates among the indoles target many receptors, for example PDE-receptors e.g. by tadalafil, 5-HT receptors e.g. by naratriptane and HMG-CoA reductases e.g. by fluvastatin (Wink, 2000; de Sa et al., 2009). Several indole alkaloids have stimulant and hallucinogenic properties (Wink, 2000; Wink and van Wyk, 2008).

As the nitrogen of steroidal alkaloids does not originate from amino acids they belong to pseudoalkaloids. A member of steroidal alkaloids is the teratogenic cyclopamine, which can cause cyclopean eyes in vertebrates (Roberts and Wink, 1998; Incardona et al., 1998).

Piperidine and diketopiperazine are six-membered non-aromatic moieties in alkaloids whereby diketopiperazine is a cyclic dipeptide having two oppositely located nitrogen. Pyrazine has the same position of nitrogen, though aromatic. Here we also can find medicinal use of active ingredients such as the oxytocin antagonist retosiban and plinabulin which is still in clinical trial against multiple drug resistant non-small cell lung cancer (Borthwick and Liddle, 2011; Mohanlal et al., 2019).

Tropane alkaloids are widespread and their plants one of the oldest medicines to use because of spasmolytic, mydriatic and hallucinogenic properties (Wink and van Wyk, 2008; van Wyk and Wink, 2017). They contain a special bicyclic moiety which is made of a seven-membered ring and a nitrogen atom which is linked to its C-1 and C-5 and forms the second ring (Osman et al., 2013). Due to their spasmolytic effect nowadays we use tropane alkaloids such as scopolamine or atropine for digestive tract spastic conditions and for ophthalmological purposes (Kukula-Koch and Widelski, 2017).

The most popular alkaloid caffeine belongs to purine alkaloids, which are consumed by many people on all continents. Theobromine, theophylline and caffeine are common members found as main ingredients in chocolate, mate, cola, green and black tea or coffee (Baumann and Frischknecht, 1988). By inhibiting adenosine receptors and cAMP phosphodiesterase they can mediate a stimulant effect (van Wyk and Wink, 2017).

**Figure 2:**
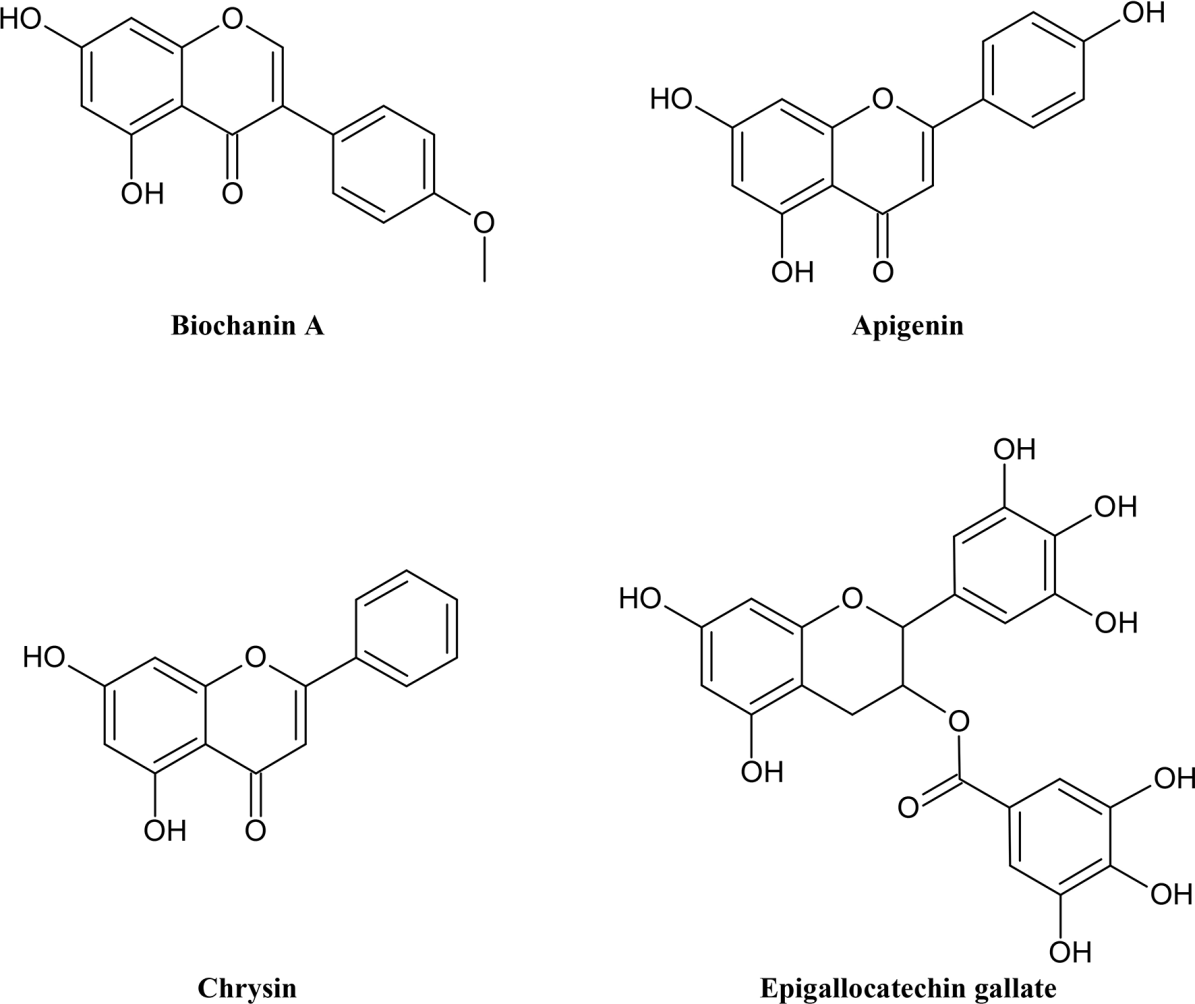
Chemical structures of some selected flavonoids with MDR reversal effects.

Flavonoids (**Figure 2**) are another complex but also often colored group of secondary metabolites. Unlike alkaloids, we can make a general statement about their common origin and basic skeleton. Flavonoids can be classified as polyphenols which share a common biosynthesis. They contain aromatic rings with phenolic hydroxyl groups. These phenolic hydroxyl groups can dissociate under physiological condition and form negatively charged phenolate ions. Because of these properties flavonoids and polyphenols can interact with proteins forming multiple hydrogen and ionic bonds (Wink, 2015). Flavonoids are widely distributed in plants and are responsible, inter alia, for their pollinator attracting colors, ultraviolet light protection, antioxidant and antimicrobial functions, and mediating symbiosis with bacteria. Including the main subgroups flavones, isoflavones, flavonols, flavanones, anthocyanins, chalcones and catechins, they derive from flavan, a benzopyran structure with a phenyl ring in position 2. Flavonoids with a phenyl ring in position 3 and 4 are called iso- and neoflavonoids, respectively. The variety of flavonoids comes from many functional groups and different states of oxidation of the heterocycle (Koes et al., 1994; Wink and van Wyk, 2008; Hänsel and Sticher, 2009). Although there has been a lot of research on the antioxidant capacity of flavonoids, the mechanism is not fully understood yet. Many studies have reported anti-inflammatory, anti-carcinogenic, anti-mutagenic, antiviral, anti-allergic and osteogenetic potentials of flavonoids in vitro. There is evidence, that polyphenols are also important for the pharmacological activity of many medicinal plants (van Wyk and Wink, 2017). Still there is a lack of information about how the necessary bioavailability is achieved in the human body as polyphenols are polar compounds (Panche et al., 2016). Most common representatives in food are luteolin and apigenin. Isoflavones are known for their estrogenic properties (Wink, 2015).

**Figure 3:**
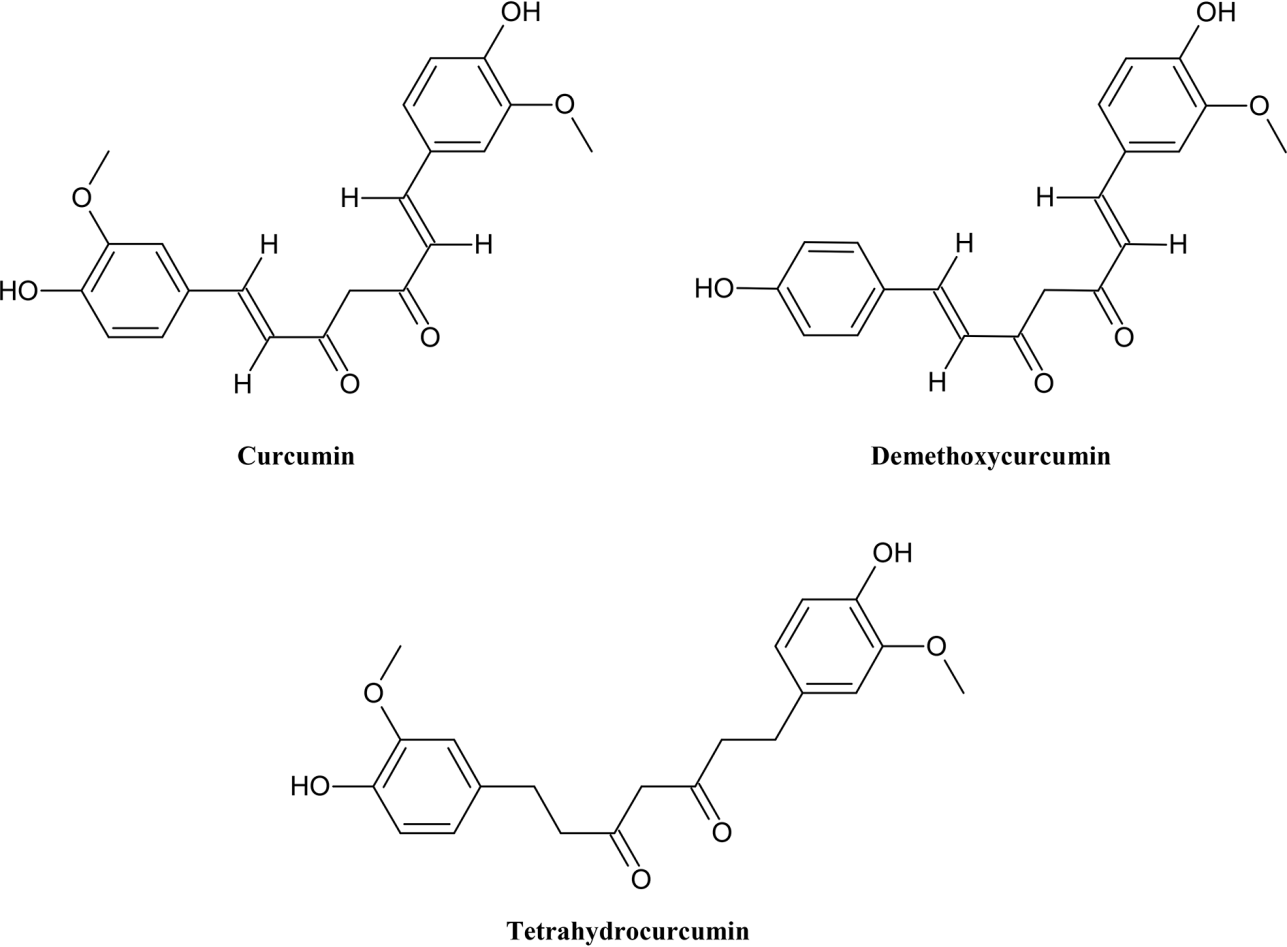
Chemical structures of some selected curcuminoids with MDR reversal effects.

In addition to flavonoids, there are smaller but also important groups of polyphenols, for example stilbenoid with resveratrol as its main member known for its potential as anti-cancer (Huang et al., 2014), antioxidant and anti-aging agent (Alamolhodaei et al., 2017). Curcuminoids (**Figure 3**) have been widely studied and have been found to have many functions such as antioxidative, anti-cancer, anti-microbial and anti-inflammatory effects in humans. They can interact with many targets (Fantini et al., 2015).

**Figure 4:**
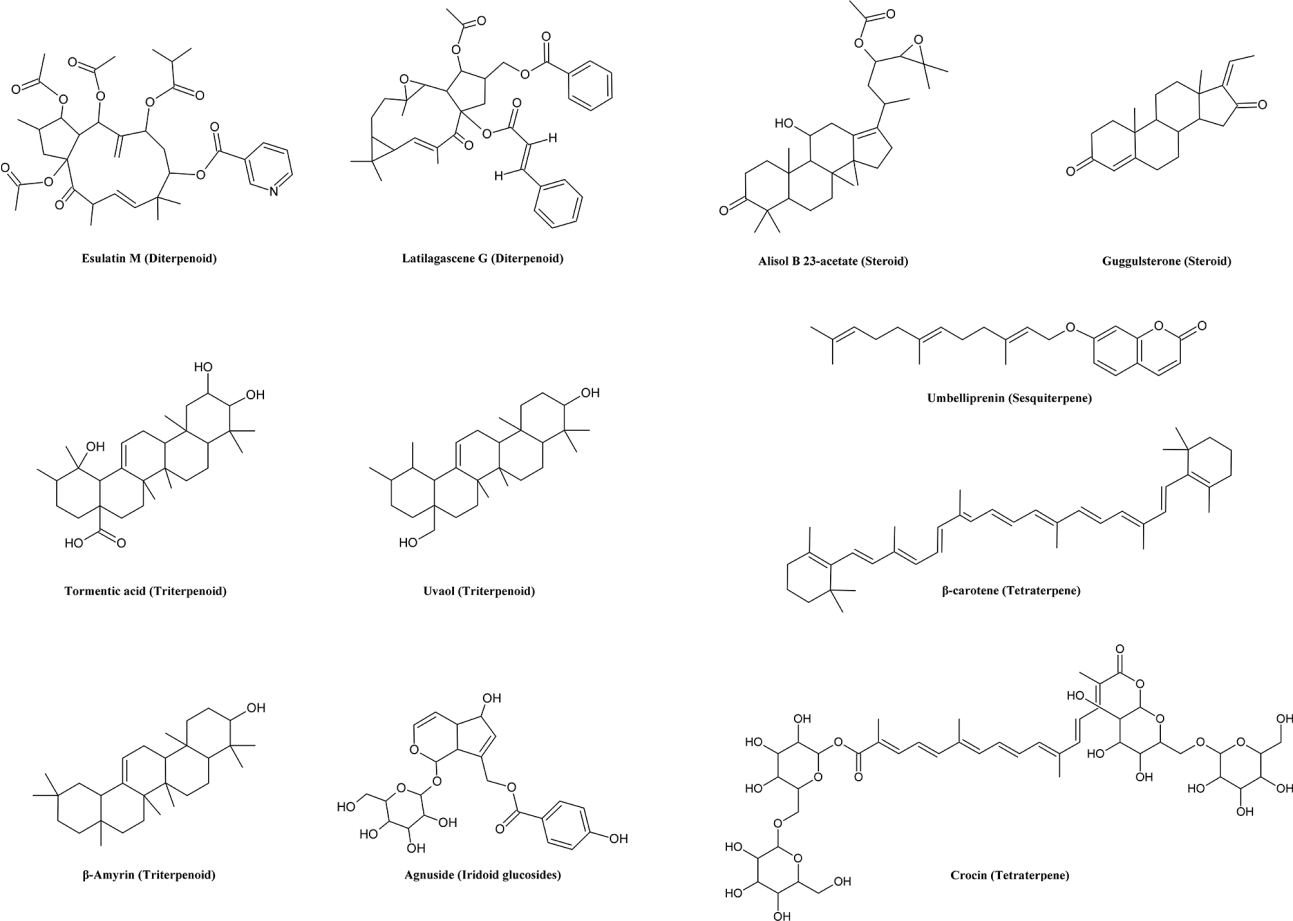
Chemical structures of some selected terpenes with MDR reversal effects.

Terpenes (**Figure 4**) are widely distributed in plants, fungi and animals. They are composed of different numbers of isoprene units, forming mono- (C10), di- (C20), sesqui- (C15), tetra- (C40) and triterpenes (C30). Many mono- and sequiterpenes are volatile and aromatic and typical ingredient of essential oils. These compounds are often lipophilic and can thus modulate the fluidity and permeability of biomembranes in animals and microbes. Many plants with essential oil have been used in traditional medicine for treatment of microbial infections and inflammation (Wink, 2015; van Wyk and Wink, 2017).

Known for their skin permeation enhancing ability, terpenes have been used as moieties of synthetic structures for topical use (Smith and Maibach, 1995). In vitro studies have shown anti-cancer, antimicrobial and antioxidant activities but for practice and use in humans this class of secondary metabolites must be further investigated (Lu et al., 2012; Cör et al., 2018). Squalene is a standard triterpene produced in plants and animals, and it is the precursor for steroid synthesis. Glycosides of steroids or triterpenes, so-called saponins have one or more polysaccharides attached saponins are amphiphilic and react as a detergent. They generally form stable foams and complex cholesterol in biomembranes. As a consequence, saponins can lyse biomembranes (Bloch, 1983). In traditional medicine they have been used for example as expectorants and anti-infectants (Wink, 2015). As effective components of vaccine adjuvants they enhance the cellular immune response (Sun et al., 2009).

Carotenoids are tetraterpenes with many conjugated double bonds. They often exhibit yellow to purple colors and can function as anti-oxidants and precursors for vitamin A.

As the name implies polyketides contain carbonyl groups positioned between methylene groups. Still they vary in shape and volume. Although they appear with an impressive structural variety, they have their source from the same biosynthetic pathway. The most famous members among drugs may be the antibiotic erythromycin or the antifungal amphotericin B.

### Phytochemicals Modulating MDR Targeting ABC-Transporters

A considerable number of secondary metabolites, which affect ABC transporters has already been discovered (**Table 1**) and several of them will be discussed in the following; glaucine (an isoquinoline alkaloid) increased the efflux of substrates such as ADR and MTX in the P-gp over-expressing cell lines of MCF-7/ADR and reduced DOX resistance in Caco-2 and CEM/ADR5000 (Eid et al., 2013; Lei et al., 2013). Tetrandrine (a benzolisoquinoline alkaloid) also caused an inhibition of efflux in Caco-2 and CEM/ADR5000 cells (Sun and Wink, 2014). A 5-substituted derivative of it named PY35 was tested for the MDR reversal activity, and showed more MDR reversal than the natural compound in resistant K562/ADM and MCF-7/ADM cells (Cao et al., 2014). Hernandezine, a bisbenzyl-isoquinoline, is a potent inhibitor of P-gp in MDR19-HEK293 cells and was able to resensitize MDR19-HEK293 and KB-V-1 cells to DOX after entering the cell membrane (Hsiao et al., 2016). High MDR-reversing activities of the quinoline derivatives were linked to the presence of two aryl rings in the hydrophobic moiety, deviation of the aryl rings from a common plane, basicity of nitrogen atom in piperazine, as well as to the distance between the hydrophobic moiety and the basic nitrogen of piperazine which must be no less than 5 Å (Suzuki et al., 1997). The quinolyl group was also suggested to have a key role in the activity of quinolines, because substitution of a quinoline ring by a naphthyl ring or a phenyl ring resulted in the reduction of MDR-reversing activity of the compounds (Suzuki et al., 1997). Among indole alkaloids, antofine showed synergistic effects with PTX in A549-PA cells and overcame resistance to PTX (Kim et al., 2012). The β-carboline harmine reversed the resistance of MTX and CPT in MDA-MB-231 cells with BCRP as overexpressed transporter but it could not affect the P-gp over-expressing CEM/ADR5000 cells the same way in this study (Ma and Wink, 2010). Not only has harmine been tested in combination with DOX but also as three-drug-combination with DTN both of which showed here an increase of effect on cells and a reduction of DOX resistance in Caco-2 and CEM/ADR5000 cells (Eid et al., 2013). As an N-acylpiperidine, piperine inhibited the efflux of the tested substrate which led to an increase of its concentration in Caco-2 and CEM/ADR5000 cells (Li et al., 2018a). Tests of piperine on MCF-7/DOX and A-549/DDP cells resulted in an increase in cytotoxicity of MTX and DOX (Li et al., 2011). Gravacridonetriol, gravacridonediol and its monomethyl ether, all had an inhibitive effect on P-gp L5178/MDR1 cells, which also led to a higher cytotoxicity of DOX (Rethy et al., 2008). Substitution of methyl groups at the positions C-2 and C-4 of acridone led to the increased lipophilicity, which can enhance the binding affinity of acridone derivatives to P-gp (Mayur, 2015). Murahari et al. (2017) studied on 2,4-dimethylacridone derivatives and showed that these compounds were potential modulators of P-gp-mediated MDR. 2,4-dimethylacridones are tricyclic and hydrophobic and have methyl groups at C-2 and C-4 positions with a propyl or butyl side chain containing terminally substituted tertiary amino groups. They found that an alkyl side chain and hydroxyl substituted secondary amine are necessary for the acridones to reverse the P-gp-mediated multidrug resistance. Moreover, an alkyl side chain of length four (butyl) was found to have higher biological activity (Murahari et al., 2017). Hegde et al. (2004) reported that replacing the hydrogen atom at position C-4 by a methoxy group slightly enhanced the lipophilicity of the acridone derivatives. Furthermore, they investigated the MDR-reversing activity of N-10-substituted acridones and N-10-substituted 4-methoxyacridones relative to their corresponding unsubstituted counterparts where the C-4 positions of the acridone rings are occupied by a hydrogen atom and a methoxy group, respectively. The parent acridone and 4-methoxyacridone had the least effect in inhibiting drug efflux suggesting that N-10-substitution is necessary for an ideal activity. It was also found that 4-methoxyacridone derivatives are more efficient than their acridone derivatives' counterparts in increasing drug accumulation. Several N-10-substituted acridones and N-10-substituted-4-methoxyacridones showed MDR-reversing activity greater than the P-gp inhibitor verapamil (Hegde et al., 2004). In another study eighteen N-10-substituted-2-bromoacridones were examined for the anti-MDR activity and compared to the parent compound 2-bromo-10H-acridin-9-one. N-10-substitution was suggested to be necessary for optimal activity of 2-bromoacridones because the parent compound had the least effect in efflux inhibiting activity (Mayur et al., 2006).

**Table 1:** Phytochemicals modulating MDR via ABC-transporters.

The effects of secondary metabolites on different cell lines expressing ABC-transporters – Transporters targeted directly					
	Substance	Cell line	Assay system	Result	Reference
**Alkaloids**					
Quinolines, Isoquinolines, Quinazolines	Dauriporphine	MES-SA/DX5 and HCT15	MDR reversing activity (cytotoxicity assay in the presence and absence of an anticancer drug, PTX)	Increase in cytotoxicity of PTX (inhibition of P-gp MDR)	Min et al. (2006)
	Fangchinoline	MDR1-MDCK II	MDR reversing activity	Decrease in substrate (PTX) efflux, inhibition of the multidrug resistance of antitumor drug PTX	He et al. (2010)
	Glaucine	Caco-2, CEM/ADR5000	MTT assay, Rh-123 accumulation assay using fluorospectroscopy	Reversal of DOX resistance in both cell lines with very high effect, synergism with DOX	Eid et al. (2013)
		Caco-2, CEM/ADR5000	MTT assay, Rh-123 accumulation assay using fluorospectroscopy	Sensitization of cell lines, enhancement of cytotoxicity, strong reduction of the IC_50_ value of DOX and consequently increase of efficacy, synergism with DOX and DTN	Eid et al. (2013)
		MCF-7/ADR	MDR reversing activity, ADR and MTX efflux assay, real-time RT-PCR, P-gp and MRP1 ATPase activity assay	Inhibition of P-gp and *MRP1*-mediated efflux, suppression of the expression of *MDR1* and *MRP1* genes, reversion of the resistance of MCF-7/ADR to ADR and MTX, increase in P-gp and MRP1 ATPase activities	Lei et al. (2013)
	Hernandezine	MDR19-HEK293, KB-V-1, NCI-ADR-RES	MDR reversing activity, fluorescent drug (calcein-AM and pheophorbide A) accumulation assay,	Inhibition of the transport function of ABCB1 (P-gp), increase in calcein-AM accumulation in MDR19-HEK293 cells, resensitizing of MDR19-HEK293 cells to DOX, resensitizing of KB-V-1 cells to DOX, colchicine and VCR, resensitizing of NCI-ADR-RES cells to DOX, colchicine and vincristine	Hsiao et al. (2016)
	Roemerine	MDR KB-V1	Cytotoxicity assay with VBL	Cytotoxicity synergism	You et al. (1995)
	Sanguinarine	Caco-2, CEM/ADR5000	MTT assay, Rh-123 accumulation assay using fluorospectroscopy	Reversal of DOX resistance in both cell lines with very high effect, synergism with DOX	Eid et al. (2013)
		Caco-2, CEM/ADR5000	MTT assay, Rh-123 accumulation assay using fluorospectroscopy	Sensitization of cell lines to DOX	Eid et al. (2013)
	Tetrandrine	Caco-2, CEM/ADR5000	Rh-123 accumulation	Increased accumulation of substrate, inhibited efflux in both cell lines, reduction of P-gp expression	Sun and Wink (2014)
	Quinidine homodimer	MCF-7/DX1	Radioactive substrate ([^3^H]PTX) accumulation assay, flow cytometric accumulation assay, confocal microscopy	Inhibition of the efflux of Rh-123, DOX, BODIPY-FL-prazosin, PTX and MTX,	Pires et al. (2009)
Steroidal alkaloids	Verabenzoamine	L5178/*MDR1* (human *MDR1*-gene-transfected mouse lymphoma cells)	Flow cytometric assay of Rh-123 accumulation	Increase in accumulation	Ivanova et al. (2011)
	Veralosine	L5178/*MDR1*	Flow cytometric assay of Rh-123 accumulation	Increase in accumulation	Ivanova et al. (2011)
	Veralosinine + Veranigrine	L5178/*MDR1*	Flow cytometric assay of Rh-123 accumulation	Increase in accumulation	Ivanova et al. (2011)
Indoles and β-carbolines	Antofine	A549-PA (PTX-resistant human lung cancer cell line)	P-gp expression using western blot, *MDR-1* mRNA expression using RT-PCR, Rh-123 accumulation by FACS	Reduction of P-gp and *MDR-1* mRNA expression, increase in intracellular Rh-123 content, synergism with PTX	Kim et al. (2012)
	Arboloscine	KB/VJ300	MDR reversing activity	Moderate to weak activity in reversing MDR	Gan et al. (2014)
	Conoduramine	KB-V1	Binding assay (VBL binding to KB-V1 vesicles)	Inhibition of drug-binding, resulting in circumventing multi-drug resistance	You et al. (1994)
	Coronaridine	KB-V1	Binding assay (VBL binding to KB-V1 vesicles)	Inhibition of drug-binding resulting in circumventing multi-drug resistance	You et al. (1994)
	Harmine	MDA-MB-231 (BCRP), CEM/ADR5000 (P-gp)	Rh-123 accumulation assay, cytotoxicity assay using MTT, MTX efflux assay	Reversal of MTX and CPT resistance in cell line with BCRP-mediated efflux, no effect on P-gp mediated efflux	Ma and Wink (2010)
		Caco-2, CEM/ADR5000	MTT assay	Reversal of DOX resistance in both cell lines, synergism with DOX	Eid et al. (2013)
		Caco-2, CEM/ADR5000	MTT assay, Rh-123 accumulation assay using fluorospectroscopy	Sensitization of cell lines to DOX	Eid et al. (2013)
	Kopsamine	KB/VJ300	MDR reversing activity	Appreciable activity in reversing MDR	Kam et al. (1998)
	Kopsiflorine	KB/VJ300	MDR reversing activity	Appreciable activity in reversing MDR	Kam et al. (1998)
	Lahadinine A	KB/VJ300	MDR reversing activity	Appreciable activity in reversing MDR	Kam et al. (1998)
	Leuconicine (types A - E)	KB/VJ300	MDR reversing activity	Reversal of multidrug resistance	Gan et al. (2009)
	11-Methoxykopsilongine	KB/VJ300	MDR reversing activity	Appreciable activity in reversing MDR	Kam et al. (1998)
	N-methoxycarbonyl-11,12-methylene-dioxykopsinine	KB/VJ300	MDR reversing activity	Appreciable activity in reversing MDR	Kam et al. (1998)
	Pleiocarpine	KB/VJ300	MDR reversing activity	Appreciable activity in reversing MDR	Kam et al. (1998)
	Reserpine	CEM/VLB100	MDR reversing activity	Increase in cytotoxicity, synergism with VBL	Pearce et al. (1989)
	Tryptanthrin	Caco-2	Transport across the Caco-2 cell monolayers, *MDR1* & *2* gene expression	Decrease in the efflux transport of the P-gp and MRP2 substrates (potential inhibitor of P-gp and MRP2)	Zhu et al. (2011)
	Vocamine	KB-V1	Binding assay (VBL binding to KB-V1 vesicles)	Inhibition of drug-binding resulting in circumventing multi-drug resistance	You et al. (1994)
	Yohimbine	CEM/VLB100	MDR reversing activity	Increase in cytotoxicity synergism with VBL	Pearce et al. (1989)
Piperidines, Pyrazines, Diketopiperazines	11E-didehydrostemofoline, 11Z-didehydrostemofoline	K562/Adr	MTT assay; fluorescent substrates accumulation assays	Increase in sensitivity to DOX and PTX; increase in the intracellular concentrations of Rh-123 and calcein-AM	Umsumarng et al. (2017)
	Isostemofoline	K562/Adr	MTT assay; fluorescent substrates accumulation assays	Increase in sensitivity to DOX and PTX; increase in the intracellular concentrations of Rh-123 and calcein-AM	Umsumarng et al. (2017)
	Lobeline	Caco-2, CEM/ADR5000	Rh-123 accumulation assay, cytotoxicity assay using MTT, MTX efflux assay	Inhibition of P-gp mediated efflux, accumulation of Rh-123, increase of DOX sensitivity of both cell lines	Ma and Wink (2008)
	Oxystemokerrine	KB-V1	MDR reversing activity	Slight increase in sensitivity	Chanmahasathien et al. (2011)
	Piperine	MCF-7/DOX, A-549/DDP	MDR reversing activity, Rh-123 accumulation assay, MTX efflux assay, RT-PCR	Reversal of resistance, decrease in ABCB1 and ABCG2 genes expression in MCF-7/DOX cells, decrease in ABCC1 gene expression in A-549/DDP cells	Li et al. (2011)
		Caco-2, CEM/ADR 5000	Cytotoxicity assay using MTT, Rh-123 and calcein-AM retention assay	Substrate, synergistic enhancement of cytotoxicity, inhibition of efflux and consequently accumulation of Rh-123 and calcein-AM	Li et al. (2018a)
	Stemocurtisine	KB-V1	MDR reversing activity	Slight increase of sensitivity	Chanmahasathien et al. (2011)
	Stemofoline	KB-V1	MDR reversing activity	Increase in cell sensitivity	Chanmahasathien et al. (2011)
	Tetramethylpyrazine	MCF-7/Dox	Flow cytometric evaluation of DOX accumulation, P-gp expression	Inhibition of efflux, decrease in P-gp expression	Zhang et al. (2012)
Tropane alkaloids	Pervilleine A	MDR KB-V1	Intracellular VBL accumulation, cytotoxicity	Cytotoxicity synergism with VBL, increase in VBL accumulation	Mi et al. (2001)
	Pervilleine B, C	MDR KB-V1	Cytotoxicity assay	Cytotoxicity synergism with VBL	Mi et al. (2002)
Acridone alkaloids	Acrimarine E	K562/R7	DNR accumulation assay	Inhibition of P-gp-mediated drug efflux	Bayet et al. (2007)
	Gravacridonediol	L5178/*MDR1*	Rh-123 accumulation assay, MTT assay, *MDR1* mRNA expression	Increase in Rh-123 accumulation, increase in DOX toxicity (synergism)	Rethy et al. (2008)
	Gravacridonediol monomethyl ether	L5178/*MDR1*	Rh-123 accumulation assay, MTT assay, *MDR1* mRNA expression	Increase in Rh-123 accumulation, cytotoxicity, synergism with DOX, decrease in *MDR1* mRNA	Rethy et al. (2008)
	Gravacridonetriol	L5178/*MDR1*	Rh-123 accumulation assay, MTT assay, *MDR1* mRNA expression	Increase in Rh-123 accumulation, cytotoxicity, synergism with DOX, decrease in *MDR1* mRNA	Rethy et al. (2008)
	2-Methoxycitpressine I	K562/R7	DNR accumulation assay	Inhibition of p-glycoprotein-mediated drug efflux	Bayet et al. (2007)
	Rutacridone	L5178/*MDR1*	Rh-123 accumulation assay, MTT assay, *MDR1* mRNA expression	Increase in Rh-123 accumulation	Rethy et al. (2008)
Purine alkaloids	Olomoucine II	MDCKII-ABCB1, HCT-8 and HepG2	Hoechst 33342 and DNR accumulation assay	Synergism with DNR (increase in intracellular retention of DNR)	Cihalova et al. (2013}
	Purvalanol A	MDCKII-ABCB1, HCT-8 and HepG2	Hoechst 33342 and DNR accumulation assay	Synergism with DNR (increase in intracellular retention of DNR)	Cihalova et al. (2013)
	Roscovitine	MDCKII-ABCB1, HCT-8 and HepG2	Hoechst 33342 and DNR accumulation assay	Synergism with DNR (increase in intracellular retention of DNR)	Cihalova et al. (2013)
Further alkaloids	Anandamine	HK-2	Fluorimetric measurement of the intracellular accumulation of calcein	Inhibition efflux (increase in the intracellular accumulation of calcein)	Nieri et al. (2006)
	Capsaicin	KB-C2	Determination of DNR and Rh-123 accumulation	Increase in the accumulation of DNR and Rh-123	Nabekura et al. (2005)
		Caco-2	[^3^H]-digoxin transport assay	At non-cytotoxic concentrations, inhibition of P-gp mediated efflux transport of [^3^H]-digoxin	Han et al. (2006)
		Caco-2, CEM/ADR 5000	Cytotoxicity assay using MTT, Rh-123 and calcein-AM retention assay	Substrate, synergistic enhancement of cytotoxicity, inhibition of efflux and consequently accumulation of Rh-123 and calcein-AM	Li et al. (2018a)
	Galantamine dimer	MCF-7/DX1	Measuring Rh-123 and DOX accumulation	Inhibition of efflux (increased accumulation)	Namanja et al. (2009)
**Polyphenols**					
Flavonoids	Acacetin	K562/BCRP	Cytotoxicity assay	Increase in cytotoxicity of SN-38 and MTX, strong reversing activity of BCRP-mediated drug resistances	Imai et al. (2004)
		MDA-MB-231	BCECF accumulation assay	Inhibition of the efflux of MRP1 fluorescent substrate (BCECF) from breast cancer cells	Wesołowska et al. (2009)
	Afrormosin	L5178/*MDR1*	Cytotoxicity assay, Rh-123 accumulation assay	Moderately effective on the human *MDR1*-transfected mouse lymphoma cell line	Gyémánt et al. (2005)
		MDA-MB-231	Cytotoxicity assay, BCECF-AM accumulation assay	MRP-mediated efflux pump modifiers, additive effect in combination with epirubicin	Gyémánt et al. (2005)
	Amorphigenin	L5178/*MDR1*	Cytotoxicity assay, Rh-123 accumulation assay	P-gp-mediated efflux pump modifier (strong MDR-reversal effects), strong antiproliferative effects, synergistic effects in combination with epirubicin	Gyémánt et al. (2005)
	Apigenin	MCF-7 MX100	MTX accumulation, cytotoxicity assay	Increasing accumulation and inhibition of BCRP in combination with other flavonoids e.g. biochanin A, and chrysin	Zhang et al. (2004)
		K562/BCRP	Cytotoxicity assay	Strong reversing activity of BCRP-mediated drug resistances	Imai et al. (2004)
	Ampelopsin	K562/ADR	MTT assay, P-gp expression assay using PE-labeled antibody, ADR accumulation assay	Decreasing P-gp expression, reversal of MDR to ADR, increase in cytotoxicity and the intracellular ADR accumulation	Ye et al. (2009)
	7,8-Benzoflavon	MCF-7 MX100	Topotecan accumulation studies	Inhibition of the efflux, increasing the accumulation of topotecan	Zhang et al. (2005)
		MCF-7/ADR	Daunomycin accumulation assay	Decrease of daunomycin efflux, increase in [^3^H]-daunomycin accumulation, strongly potentiated cytotoxicity of daunomycin	Chung et al. (2005)
	Biochanin A	MDA435/LCC6, MCF-7/ADR, MDA435/LCCMDR1	Daunomycin accumulation, DOX cytotoxicity	Increase in [^3^H]-daunomycin accumulation, potentiation of DOX cytotoxicity, inhibition of P-gp-mediated cellular efflux	Zhang and Morris (2003)
		Panc-1	Determination of daunomycin and VBL accumulation	Increase in accumulation of daunomycin and VBL in Panc-1 cells, inhibiting MRP1-mediated drug transport	Nguyen et al. (2003)
		MCF-7 MX100	MTX accumulation, cytotoxicity assay	Increasing accumulation and inhibiting BCRP in combination with other flavonoids e.g. apigenin or chrysin	Zhang et al. (2004)
		Caco-2	Ochratoxin A (OTA) accumulation assay	Increase in OTA accumulation, impairing OTA efflux through competitive inhibition of MRP-2 pump	Sergent et al. (2005)
	Catechin	Rats jejunal membrane in vitro	P-gp stimulation/inhibition profiles using a P-gp-dependent ATPase assay	Inhibitory effect on P-gp ATPase activity	Najar et al. (2010)
		NIH-3T3-G185	Intracellular retention of Rh-123 or LDS-751 (P-gp marker substrate)	Slight facilitation of the efflux of LDS-751 (providing a chemoprotective role)	Wang et al. (2002)
	Chalcone	Panc-1	Determination of daunomycin and VBL accumulation	Increasing the accumulation of daunomycin and VBL in Panc-1 cells, inhibiting MRP1-mediated drug transport	Nguyen et al. (2003)
	Chrysin	CaCo-2	Ochratoxin A (OTA) accumulation assay	Increase in OTA accumulation, impairing OTA efflux through competitive inhibition of MRP-2 pump	Sergent et al. (2005)
		MCF-7 MX100	MTX accumulation, topotecan accumulation studies, cytotoxicity assay	Increasing accumulation and inhibiting BCRP in combination with other flavonoids e.g. Biochanin A, increase in accumulation of topotecan (inhibition of the BCRP-mediated transport of topotecan)	Zhang et al. (2004; 2005)
		K562/BCRP	Cytotoxicity assay	Strong reversing activity of BCRP-mediated drug resistances	Imai et al. (2004)
		L5178/*MDR1*	Cytotoxicity assay, Rh-123 accumulation assay	P-gp-mediated efflux pump modifiers (increase in Rh-123 accumulation), strong antiproliferative effects, synergistic effects in combination with epirubicin	Gyémánt et al. (2005)
	Chrysosplenol D	Staphylococcus aureus	S. aureus growth inhibition assay	Synergism with berberine and norfloxacin (Potentiated the activity of berberine and norfloxacin against a resistant strain of S. aureus), MDR pump inhibitor	Stermitz et al. (2002)
	Diosmetin	K562/BCRP	Cytotoxicity assay	Strong reversing activity of BCRP-mediated drug resistances	Imai et al. (2004)
	Diosmin	Caco-2	Accumulation of Rh-123	Increase in accumulation of Rh-123	Yoo et al. (2007a)
	(+)Epicatechin	NIH-3T3-G185	Intracellular retention of Rh-123 or LDS-751 (P-gp marker substrate)	Slightly facilitated active transport (efflux) of LDS-751 (providing a chemoprotective role)	Wang et al. (2002)
	(-)Epicatechin	NIH-3T3-G185	Intracellular retention of Rh-123 or LDS-751 (P-gp marker substrate)	Significant enhance of the active transport (efflux) of LDS-751 (providing a chemoprotective role)	Wang et al. (2002)
		KB-C2	Rh-123 and DNR accumulation	No effect on P-gp efflux	Kitagawa et al. (2004)
	(-)Epicatechin gallate	NIH-3T3-G185	Intracellular retention of Rh-123 or LDS-751 (P-gp marker substrate)	Slight inhibition of LDS efflux	Wang et al. (2002)
	Epicatechin gallate	KB-C2	Rh-123 and DNR accumulation	Increase in cellular accumulation of Rh-123 and DNR	Kitagawa et al. (2004)
		Bel-7404/DOX, mouse models	Cell proliferation, Rh-123 and DOX (DOX) accumulation assay, semi-quantitative RT-PCR analysis of *MDR1* mRNA expression	At higher doses a slight inhibitory effect on cell proliferation, administration of DOX with ECG at lower doses significant inhibition of cell proliferation in vitro and hepatoma growth in a xenograft mouse model, increase in DOX and Rh-123 accumulations, decreasing P-gp in cells concurrently treated by DOX and ECG, reduction of the expression of *MDR1* mRNA in BEL-7404/DOX cells treated by DOX and ECG	Liang et al. (2010)
	(-)Epigallocatechin	NIH-3T3-G185	Intracellular retention of Rh-123 or LDS-751 (P-gp marker substrate)	Inhibition of LDS efflux	Wang et al. (2002)
	Epigallocatechin	KB-C2	Rh-123 and DNR accumulation	Increasing the accumulation of DNR	Kitagawa et al. (2004)
		MDA-MB-231	BCECF-AM accumulation assay	MRP-mediated efflux pump modifiers	Gyémánt et al. (2005)
	Epigallocatechin gallate	NIH-3T3-G185	Intracellular retention of Rh-123 or LDS-751 (P-gp marker substrate)	Slight inhibition of the efflux of Rh-123, enhancing the efflux of LDS	Wang et al. (2002)
		KB-C2	Rh-123 and DNR accumulation	Increase in Rh-123 and DNR accumulation, decrease in the efflux of Rh-123	Kitagawa et al. (2004)
		Bel-7404/DOX, mouse models	Cell proliferation, Rh-123 and DOX (DOX) accumulation assay, Semi-quantitative RT-PCR analysis of *MDR1* mRNA expression	Slight inhibitory effect on cell proliferation at higher doses, significant inhibition of cell proliferation and hepatoma growth by the administration of DOX with EGCG at lower doses in vitro in a xenograft mouse model, increasing DOX and Rh-123 accumulations, decrease of P-gp in cells concurrently treated by DOX and EGCG, expression of *MDR1* mRNA in BEL-7404/DOX cells treated by DOX and EGCG reduced	Liang et al. (2010)
		CEM/ADR5000	MTT assay, Rh-123 accumulation assay using fluorospectroscopy	Reversal of DOX resistance in the cancer cell line	Eid et al. (2013)
		Caco-2, CEM/ADR5000	MTT assay, Rh-123 accumulation assay using fluorospectroscopy	Sensitization of cell lines to DOX	Eid et al. (2013)
		MCF-7	Cytotoxicity using MTS assay, P-gp protein expression using western blot and immunofluorescence microscopy, Rh-123 accumulation	Increase in intracellular Rh-123 accumulation, decrease in P-gp protein expression, reduction of cell viability	Reyes-Esparza et al. (2015)
		Caco-2, CEM/ADR5000	Cytotoxicity determination using MTT assay after combining with DOX, Rh-123 assay and Calcein-AM assay testing P-gp activity	DOX sensitization, synergistic effect on the leukemia cell line	Li et al. (2018b)
		Caco-2, CEM/ADR5000	Cytotoxicity determination using MTT assay after combining with DOX + DTN, Rh-123 assay and Calcein-AM assay testing P-gp activity	DOX sensitization, synergism in both cell lines	Li et al. (2018b)
	Formononetin	L5178/*MDR1*	Cytotoxicity assay, Rh-123 accumulation assay	P-gp-mediated efflux pump modifier (strong MDR-reversal effects)	Gyémánt et al. (2005)
		MDA-MB-231	Cytotoxicity assay, BCECF-AM accumulation assay	MRP-mediated efflux pump modifier, synergistic effects in combination with epirubicin	Gyémánt et al. (2005)
	Genistein	K562/BCRP	Topotecan and [^3^H]Genistein accumulation, cytotoxicity assay	Increasing cytotoxicity of SN-38 and MTX, increasing accumulation of [^3^H]Genistein and topotecan, inhibition of BCRP-mediated drug efflux	Imai et al. (2004)
		Panc-1	Determination of daunomycin and VBL accumulation	Increasing the accumulation of DNM and VBL in Panc-1 cells, inhibiting MRP1-mediated drug transport	Nguyen et al. (2003)
		CaCo2	Ochratoxin A (OTA) accumulation assay	Increase in OTA accumulation, impairing OTA efflux through competitive inhibition of MRP-2 pump	Sergent et al. (2005)
	Glabridin	MDCKII	Transport of [^3^H]digoxin	Inhibition of P-gp-mediated transport of digoxin	Cao et al. (2007)
		KB-C2	DNR accumulation	Increase of DNR accumulation (inhibition of the P-gp-mediated efflux of DNR)	Nabekura et al. (2008a)
	3,3',4',5,6,7,8-Heptamethoxyflavone	K562/ADM	Uptake of [^3^H]vincristine	Increasing the uptake of [^3^H]vincristine (inhibition of P-gp mediated efflux of [^3^H]vincristine)	Ikegawa et al. (2000)
	Kaempferol	Panc-1	Determination of daunomycin and VBL accumulation	Increase in accumulation of DNM and VBL in Panc-1 cells, inhibiting MRP1-mediated drug transport	Nguyen et al. (2003)
		KB-C2	Rh-123 and DNR accumulation	Increase in the accumulation of Rh-123 and DNR (inhibition of substrate efflux)	Kitagawa et al. (2005)
		K562/BCRP	Cytotoxicity assay	Increasing cytotoxicity of SN-38 and MTX, strong reversing activity of BCRP-mediated drug resistances	Imai et al. (2004)
		MDA-MB-231	Cytotoxicity assay, BCECF-AM accumulation assay	MRP-mediated efflux pump modifier, synergistic effects in combination with epirubicin	Gyémánt et al. (2005)
	Luteolin	K562/BCRP	Cytotoxicity assay	Strong reversing activity of BCRP-mediated drug resistances	Imai et al. (2004)
	Morin	MDA435/LCC6, MCF-7/ADR, MDA435/LCCMDR1	Daunomycin (DNM) accumulation, P-gp ATPase activity assay, [^3^H]azidopine photoaffinity labeling	Increase in DNM accumulation, inhibition of P-gp ATPase activity, inhibition of P-gp-mediated cellular efflux, inhibition of [^3^H]azidopine photoaffinity labeling of P-gp suggesting a direct interaction with P-gp substrate binding	Zhang and Morris (2003)
		Panc-1	Determination of daunomycin and VBL accumulation	Increasing the accumulation of DNM and VBL in Panc-1 cells, inhibiting MRP1-mediated drug transport	Nguyen et al. (2003)
	Myricetin	MDCKII-MRP1, MDCKII-MRP2	Efflux of calcein (inhibition of MRP1 and MRP2 activity was studied using the fluorescent calcein as a model substrate	Inhibition of MRP1 and MRP2 activity (inhibition of calcein efflux)	van Zanden et al. (2005)
	Naringenin	MCF-7/ADR	[^3^H]-Daunomycin (DNM) accumulation, [^3^H]-Daunomycin efflux study	Increase in accumulation of DNM, decrease in efflux of DNM	Chung et al. (2005)
		Caco-2	The flux of talinolol across Caco-2 cell monolayers	Reduction of P-gp mediated secretory transport of talinolol (inhibition of P-gp)	De Castro et al. (2008)
		K562/BCRP	Cytotoxicity assay, topotecan accumulation	Increase in cytotoxicity of SN-38 and MTX, increase in accumulation of topotecan	Imai et al. (2004)
	Naringenin-7-glucosid	K562/BCRP	Cytotoxicity assay	Inhibition of BCRP-mediated drug resistance	Imai et al. (2004)
	Nobiletin	KB-C2, KB/MRP	Accumulation assay of DNR in KB-C2 cells and calcein in KB/MRP cells, P-gp ATPase activity	Increase in the accumulation of DNR in KB-C2 cells, increase in calcein accumulation in KB/MRP cells, stimulation of ATPase activity of P-gp	Nabekura et al. (2008b)
		K562/ADM	Uptake of [^3^H]vincristine	Increase in the uptake of [^3^H]vincristine (inhibition of P-gp mediated efflux of [^3^H]vincristine)	Ikegawa et al. (2000)
		A2780/T, A549/T	Cytotoxicity assay (SRB assay); intracellular accumulation of Rh-123, DOX and Flutax-2 using flow cytometry	Sensitization of cells to chemotherapeutic drugs PTX, DOX, DNR and docetaxel; synergism with PTX; increase in intracellular accumulation of Rh-123, DOX and Flutax-2	Ma et al. (2015)
	Phloretin	MDA435/LCC6, MCF-7/ADR	Daunomycin (DNM) accumulation	Increase in DNM accumulation, inhibition of P-gp-mediated cellular efflux	Zhang and Morris (2003)
		Panc-1	Determination of daunomycin and VBL accumulation	Increase in the accumulation of DNM and VBL in Panc-1 cells, inhibiting MRP1-mediated drug transport	Nguyen et al. (2003)
		Mouse lymphoma/MDR1 cells	Rh-123 accumulation	Moderate inhibition of efflux	Molnár et al., 2010 (review)
	Procyanidine	Rat brain microvessel endothelial cells (RBMECs)	Rh-123 intracellular accumulation assay, Rh-123 efflux assay, P-gp ATPase activity measurement	Increase in the accumulation of Rh-123, decrease in Rh-123 efflux, inhibition of the P-gp ATPase activity	He et al. (2009)
	Quercetin	Panc-1	Determination of daunomycin and VBL accumulation	Increase in the accumulation of DNM and VBL in Panc-1 cells, inhibition of MRP1-mediated drug transport	Nguyen et al. (2003)
		CaCo2	Ochratoxin A (OTA) accumulation assay	Increase of OTA accumulation, impairing OTA efflux through competitive inhibition of MRP-2 pump	Sergent et al. (2005)
		BEL/5-FU	Cytotoxicity assay; Rh-123 and ADR accumulation using flow cytometry; ABCB1, ABCC1, ABCC2 mRNA and protein expression using real-time PCR and western blot	Increase in sensitivity to chemotherapeutic drugs 5-FU, MMC and ADR; increase in intracellular Rh-123 and ADR accumulation; decrease in ABCB1, ABCC1 and ABCC2 mRNAs and proteins expression	Chen et al. (2018)
	Robinetin	MDCKII-MRP1, MDCKII-MRP2	Efflux of calcein (inhibition of MRP1 and MRP2 activity was studied using the fluorescent calcein as a model substrate	Inhibition of MRP1 and MRP2 activity (inhibited calcein efflux)	van Zanden et al. (2005)
	Robinin	MDA-MB-231	Cytotoxicity assay, BCECF-AM accumulation assay	MRP-mediated efflux pump modifiers, additive effects in combination with epirubicin	Gyémánt et al. (2005)
	Rotenone	Mouse lymphoma/MDR1, COLO320/MDR1	Rh-123 accumulation	Inhibited efflux, increased Rh-123 accumulation	Molnár et al., 2010 (review)
		L5178/*MDR1*	Cytotoxicity assay, Rh-123 accumulation assay	P-gp-mediated efflux pump modifier (MDR modulating activity), strong antiproliferative effects, additive effects in combination with epirubicin	Gyémánt et al. (2005)
	Silymarin	MCF-7/ADR	[^3^H]-Daunomycin (DNM) accumulation, [^3^H]-Daunomycin efflux study	Increase in the accumulation of DNM, decrease in efflux of DNM	Chung et al. (2005)
		MDA435/LCC6, MCF-7/ADR, MDA435/LCCMDR1	Daunomycin (DNM) accumulation, P-gp ATPase activity assay, [^3^H]azidopine photoaffinity labeling	Increase in DNM accumulation, inhibition of P-gp ATPase activity, inhibition of P-gp-mediated cellular efflux, inhibition of [^3^H]azidopine photoaffinity labeling of P-gp suggesting a direct interaction with the P-gp substrate binding	Zhang and Morris (2003)
		Panc-1	Determination of daunomycin and VBL accumulation	Increase in the accumulation of DNM and VBL in Panc-1 cells, inhibition of MRP1-mediated drug transport	Nguyen et al. (2003)
	Tangeretin	K562/ADM	Uptake of [^3^H]vincristine	Increase in the uptake of [^3^H]vincristine (inhibition of P-gp mediated efflux of [^3^H]vincristine)	Ikegawa et al. (2000)
Stilbenoids	Resveratrol	KB-C2	Determination of DNR and Rh-123 accumulation	Increase in the accumulation of DNR, decrease in the efflux of Rh-123	Nabekura et al. (2005)
		CaCo2	Ochratoxin A (OTA) accumulation assay	Increase of OTA accumulation, impairing OTA efflux through competitive inhibition of MRP-2 pump	Sergent et al. (2005)
		Caco-2, CEM/ADR5000	Cytotoxicity assay using MTT, Rh-123 accumulation assay	Increase in Rh-123 accumulation, enhancement of DOX cytotoxicity	El-Readi et al. (2019)
Curcuminoids	Curcumin	SGC7901/VCR	Analysis of apoptosis by propidium iodide (PI)-stained flow cytometry (FCM) and a morphological assay using acridine orange (AO)/ethidium bromide (EB) dual staining, accumulation and efflux of Rh123 as measured by flow cytometry, expression of P-gp by FCM using fluorescein isothiocyanate (FITC)-conjugated anti-P-gp	Promotion of VCR-mediated apoptosis, increase in Rh-123 accumulation and inhibition of the efflux of Rh-123, downregulation of P-gp expression	Tang et al. (2005)
		KBV20C	VCR, PTX	Increasing cytotoxicity	Um et al. (2008)
		KB-C2	Determination of DNR and Rh-123 accumulation	Increase in the accumulation of DNR and Rh-123	Nabekura et al. (2005)
		Caco-2, LLC-PK1, LLC-GA5-COL300	DNR transport (apical to basolateral (a-b) and basolateral to apical (b-a)) across Caco-2 cell monolayers, calcein-AM uptake in LLC-PK1 and LLC-GA5-COL300 cells	Decrease in the efflux ratio of DNR, increase of calcein-AM accumulation in LLC-GA5-COL300	Ampasavate et al. (2010)
		Caco-2, CEM/ADR5000	Cytotoxicity determination using MTT assay after combining with DOX, Rh-123 assay and Calcein-AM assay testing P-gp activity	DOX sensitization, synergistic effect on the colon cancer cell line	Li et al. (2018b)
		Caco-2, CEM/ADR5000	Cytotoxicity determination using MTT assay after combining with DOX + DTN, Rh-123 assay and Calcein-AM assay testing P-gp activity	DOX sensitization, synergism in both cell lines	Li et al. (2018b)
	Bisdemethoxycurcumin	Caco-2, LLC-PK1, LLC-GA5-COL300	DNR transport (apical to basolateral (a-b) and basolateral to apical (b-a)) across Caco-2 cell monolayers, calcein-AM uptake in LLC-PK1 and LLC-GA5-COL300 cells	Decrease in the efflux ratio of DNR	Ampasavate et al. (2010)
	Demethoxycurcumin,	Caco-2, LLC-PK1, LLC-GA5-COL300	DNR transport (apical to basolateral (a-b) and basolateral to apical (b-a)) across Caco-2 cell monolayers, calcein-AM uptake in LLC-PK1 and LLC-GA5-COL300 cells	Decrease in the efflux ratio of DNR, increase of calcein-AM accumulation in LLC-GA5-COL300	Ampasavate et al. (2010)
	Tetrahydrocurcumin	KB-V1, MCF-7 MDR	Rh-123 and calcein-AM accumulation assay by FACS in KB-V1 cells, radiolabeled drug ([^3^H]-VBL) accumulation for MCF-7 MDR	Increase in the accumulation of Rh-123 and calcein-AM in KB-V-1 cells, increase in the accumulation and inhibition of the [^3^H]-VBL efflux in MCF-7 MDR	Limtrakul et al. (2007)
Others	Tannic acid	Caco-2, CEM/ADR5000	Cytotoxicity determination using MTT assay after combining with DOX, Rh-123 assay and Calcein-AM assay testing P-gp activity	DOX sensitization, synergistic effect on the colon cancer cell line	Li et al. (2018b)
		Caco-2, CEM/ADR5000	Cytotoxicity determination using MTT assay after combining with DOX + DTN, Rh-123 assay and Calcein-AM assay testing P-gp activity	DOX sensitization, synergism in both cell lines	Li et al. (2018b)
**Phenylpropanoids**					
**Neo-/lignans**	Arctigenin	Caco-2	Cytotoxicity determination using MTT assay after combining with DOX, Rh-123 accumulation assay testing P-gp activity	Synergism in Caco-2 cells and slight synergistic effect in CEM/ADR5000, increase in Rh-123 accumulation	Su et al. (2015)
		Caco-2, CEM/ADR5000	Cytotoxicity determination using MTT assay after combining with DOX + DTN, Rh-123 accumulation assay testing P-gp activity	Significant increase in synergism of combination with DOX by DTN, increase in Rh-123 accumulation	Su et al. (2015)
	Arctiin	Caco-2, CEM/ADR 5000	Cytotoxicity determination using MTT assay after combining with DOX, Rh-123 accumulation assay testing P-gp activity	Moderate synergistic effect in CEM/ADR (concentration-dependent) and in Caco-2 cells, increase in Rh-123 accumulation	Su et al. (2015)
			Cytotoxicity determination using MTT assay after combining with DOX + DTN, Rh-123 accumulation assay testing P-gp activity	Significant increase in synergism of combination with DOX by DTN, increase in Rh-123 accumulation	Su et al. (2015)
	Epimagnolin A	Flp-In-293/ABCB1	MTT assay; calcein assay	Enhancement of sensitivity to anti-cancer drugs DNR, DOX, VBL and VCR; inhibition of calcein efflux	Mitani et al. (2018)
	Iso-/lappaol A	Caco-2	Cytotoxicity determination using MTT assay after combining with DOX, Rh-123 accumulation assay testing P-gp activity	Slight synergistic effect in Caco-2 cells, additive effect in CEM/ADR cells, increase in Rh-123 accumulation	Su et al. (2015)
		Caco-2, CEM/ADR5000	Cytotoxicity determination using MTT assay after combining with DOX + DTN, Rh-123 accumulation assay testing P-gp activity	Significant increase in synergism of combination with DOX by DTN, increase in Rh-123 accumulation	Su et al. (2015)
	Lappaol C	Caco-2, CEM/ADR 5000	Cytotoxicity determination using MTT assay after combining with DOX, Rh-123 accumulation assay testing P-gp activity	Synergistic effect in CEM/ADR (concentration-dependent) and in Caco-2 cells	Su et al. (2015)
		Caco-2, CEM/ADR 5000	Cytotoxicity determination using MTT assay after combining with DOX + DTN, Rh-123 accumulation assay testing P-gp activity	Significant increase in synergism of combination with DOX by DTN	Su et al. (2015)
	Lappaol F	Caco-2, CEM/ADR 5000	Cytotoxicity determination using MTT assay after combining with DOX, Rh-123 accumulation assay testing P-gp activity	Moderate synergism in CEM/ADR 5000 cells with concentration-dependent activity, stronger effect in Caco-2 cells, increase in Rh-123 accumulation	Su et al. (2015)
			Cytotoxicity determination using MTT assay after combining with DOX + DTN, Rh-123 accumulation assay testing P-gp activity	Significant increase in synergism of combination with DOX by DTN, increase in Rh-123 accumulation	Su et al. (2015)
	Matairesinol	KB-C2, KB/MRP	DNR and calcein accumulation assay	Increasing accumulation of DNR (inhibits the P-gp-mediated efflux of DNR) and calcein (inhibits the MRP1-mediated efflux of calcein)	Nabekura et al. (2008a)
		Caco-2, CEM/ADR 5000	Cytotoxicity determination using MTT assay after combining with DOX, Rh-123 accumulation assay testing P-gp activity	Synergistic effect in Caco-2 cells, increase in Rh-123 accumulation, reversal of multidrug resistance	Su et al. (2015)
		Caco-2, CEM/ADR 5000	Cytotoxicity determination using MTT assay after combining with DOX + DTN, Rh-123 accumulation assay testing P-gp activity	Significant increase in synergism of combination with DOX by DTN, increase in Rh-123 accumulation	
	Sesamin	KB-C2	DNR accumulation assay	Increase in DNR accumulation (inhibition of the P-gp-mediated efflux of DNR)	Nabekura et al. (2008a)
Dibenzocyclo-octadienelignans	Gomisin A	HepG2-DR	Cellular Rh-123 accumulation assay by flow cytometry, determination of P-gp-associated ATPase activity, photoaffinity labeling of P-gp with [^125^I]iodoarylazidoprazosin	Restoration of the cytotoxicity of VBL and DOX, inhibition of the P-gp ATPase activity, additive effect with verapamil and vanadate on the inhibition of Rh-123 efflux, inhibition of [^125^I]IAAP photo-crosslinking of P-gp	Wan et al. (2006)
	Schisandrin A (Deoxyschizandrin)	Caco-2	Rh-123 uptake assay, bidirectional transports of digoxin and Rh-123	Increase in Rh-123 accumulation, increase in apical-to-basal transports of digoxin and Rh-123, decrease in basal-to-apical transports	Yoo et al. (2007b)
		COR-L23/R	MTT assay, DOX accumulation assay	Restoration of the cytotoxic action of DOX to COR-L23/R cells, increase in the accumulation of DOX	Slaninová et al. (2009)
	Schisandrin B/γ-Schizandrin	COR-L23/R	MTT assay, DOX accumulation assay	Restoration of the cytotoxic action of DOX to COR-L23/R cells, increase in the accumulation of DOX	Slaninová et al. (2009)
	Schisandrol A	HepG2-DR	Flow cytometry analyses of cell cycle and Rh-123 efflux, P-gp-ATPase activity assay	Strong synergistic effect (enhanced cytotoxicity) with DOX, VBL and taxol, restoration VBL-induced G2/M arrest, increase in cellular retention of Rh-123, stimulation of basal P-gp-ATPase	Fong et al. (2007)
Others	Chlorogenic acid	Rats jejunal membrane in vitro	P-gp stimulation/inhibition profiles using a P-gp-dependent ATPase assay	Inhibitory effect on P-gp ATPase activity	Najar et al. (2010)
	Ginkgolic acid	KB-C2	DNR accumulation	Increase of DNR accumulation (inhibiting the P-gp-mediated efflux of DNR)	Nabekura et al. (2008a)
**Terpenes**					
Monoterpenes	Citronellal	LLC-GA5-COL150	Intracellular accumulation of [^3^H]digoxin	Increase in [^3^H]digoxin accumulation	Yoshida et al. (2005)
	(R)-(+)-citronellal	LLC-GA5-COL150	Intracellular accumulation of [^3^H]digoxin	Increase in [^3^H]digoxin accumulation	Yoshida et al. (2005)
	(S)-(–) β-citronellol	LLC-GA5-COL150	Intracellular accumulation of [^3^H]digoxin	Increase in [^3^H]digoxin accumulation	Yoshida et al. (2005)
	Cineole	LLC-GA5-COL150	Intracellular accumulation of [^3^H]digoxin	Increase in [^3^H]digoxin accumulation	Yoshida et al. (2005)
	DL-citronellol	LLC-GA5-COL150	Intracellular accumulation of [^3^H]digoxin	Increase in [^3^H]digoxin accumulation	Yoshida et al. (2005)
	Menthol	Caco-2, CEM/ADR5000	MTT assay, Rh-123 accumulation assay using fluorospectroscopy	Reversal of DOX resistance in both cell lines, synergism with DOX	Eid et al. (2013)
		Caco-2, CEM/ADR5000	MTT assay, Rh-123 accumulation assay using fluorospectroscopy	Synergism with DOX	Eid et al. (2013)
	Thymol	Caco-2, CEM/ADR5000	MTT assay, Rh-123 accumulation assay using fluorospectroscopy	Synergism with DOX	Eid et al. (2013)
		Caco-2, CEM/ADR5000	MTT assay, Rh-123 accumulation assay using fluorospectroscopy	Synergism with DOX	Eid et al. (2013)
Iridoid glucosides	Agnuside	Rats jejunal membrane in vitro	P-gp stimulation/inhibition profiles using a P-gp-dependent ATPase assay	Inhibitory effect on P-gp ATPase activity	Najar et al. (2010)
	Negundoside	Rats jejunal membrane in vitro	P-gp stimulation/inhibition profiles using a P-gp-dependent ATPase assay	Stimulatory effect on P-gp ATPase activity	Najar et al. (2010)
	Picroside-I	Rats jejunal membrane in vitro	P-gp stimulation/inhibition profiles using a P-gp-dependent ATPase assay	Stimulatory effect on P-gp ATPase activity	Najar et al. (2010)
	Picroside-II	Rats jejunal membrane in vitro	P-gp stimulation/inhibition profiles using a P-gp-dependent ATPase assay	Inhibitory effect on P-gp ATPase activity	Najar et al. (2010)
Sesquiterpenes	Aromadendrene	Caco-2, CEM/ADR5000	MTT assay using, Rh-123 accumulation assay using fluorospectroscopy	Synergism with DOX	Eid et al. (2013)
		Caco-2, CEM/ADR5000	MTT assay using, Rh-123 accumulation assay using fluorospectroscopy	Synergism with DOX	Eid et al. (2013)
	Farnesiferol B	MCF-7/Adr	Cytotoxicity using alamar blue assay, accumulation of Rh-123 using flow cytometry	Increase in cytotoxicity of DOX, increase in intracellular accumulation of Rh-123	Kasaian et al. (2015)
	Farnesiferol C	MCF-7/Adr	Cytotoxicity using alamar blue assay, accumulation of Rh-123 using flow cytometry	Increase in cytotoxicity of DOX, increase in intracellular accumulation of Rh-123	Kasaian et al. (2015)
	Lehmferin	MCF-7/Adr	Cytotoxicity using alamar blue assay, accumulation of Rh-123 using flow cytometry	Increase in cytotoxicity of DOX, increase in intracellular accumulation of Rh-123	Kasaian et al. (2015)
	Santonin	Rats jejunal membrane in vitro	P-gp stimulation/inhibition profiles using a P-gp-dependent ATPase assay	Inhibitory effect on P-gp ATPase activity	Najar et al. (2010)
	Umbelliprenin	MCF-7/Adr	Cytotoxicity using alamar blue assay, accumulation of Rh-123 using flow cytometry	Increase in cytotoxicity of DOX, increase in intracellular accumulation of Rh-123	Kasaian et al. (2015)
Diterpenes	Andrographolide	Rats jejunal membrane in vitro	P-gp stimulation/inhibition profiles using a P-gp-dependent ATPase assay	Biphasic effect (stimulation at low concentration and inhibition at high concentration)	Najar et al. (2010)
	Carnosic acid	KB-C2, KB/MRP	Accumulation assay of DNR and Rh-123 in KB-C2 and calcein in KB/MRP cells, ATPase activity assay, resistance to VBL cytotoxicity by a calorimetric assay	Increase in the accumulation of DNR and Rh-123 in KB-C2 cells, stimulation of the P-gp ATPase activity, sensitization of KB-C2 cells to VBL cytotoxicity	Nabekura et al. (2010)
	Carnosol	KB-C2, KB/MRP	Accumulation assay of DNR and Rh-123 in KB-C2 and calcein in KB/MRP cells, ATPase activity assay, resistance to VBL cytotoxicity by a calorimetric assay	Increase in the accumulation of DNR and Rh-123 in KB-C2 cells, stimulation of P-gp ATPase activity	Nabekura et al. (2010)
	Esulatin M	L5178Y‐MDR	MTT assay; Rh-123 accumulation assay	Synergistic interaction with DOX; increase in intracellular accumulation of Rh-123	Reis et al. (2016)
	Epoxywelwitschene	L5178Y‐MDR	MTT assay; Rh-123 accumulation assay	Synergistic interaction with DOX; increase in intracellular accumulation of Rh-123	Reis et al. (2016)
	Euphoboetirane A, C, D, E, F, G and I	L5178Y‐MDR	MTT assay; Rh‐123 accumulation assay	Increase in intracellular Rh-123 accumulation; synergism with DOX	Neto et al. (2019)
	Euphotuckeyanol	L5178/*MDR1*	Rh-123 accumulation assay, in vitro antiproliferative effect in combination with epirubicin using checkerboard microplate method	Inhibition of Rh-123 efflux (increase in accumulation), exhibition of a synergistic interaction with epirubicin and enhancement of the antiproliferative effect of epirubicin	Duarte et al. (2008)
	Euphowelwitschine A, B	L5178Y‐MDR	MTT assay; Rh-123 accumulation assay	Synergistic interaction with DOX; increase in intracellular accumulation of Rh-123	Reis et al. (2016)
	12‐Hydroxyboetirane A, B and C	L5178Y‐MDR	MTT assay; Rh‐123 accumulation assay	Increase in intracellular Rh-123 accumulation; synergism with DOX	Neto et al. (2019)
	Latilagascene A, B, C	L5178/*MDR1*	Assay for Rh-123 accumulation	Inhibition of Rh-123 efflux (increase in accumulation)	Duarte et al. (2006)
	Latilagascene G, H, I	L5178/*MDR1*	Rh-123 accumulation assay, in vitro antiproliferative effect in combination with epirubicin using checkerboard microplate method	Inhibition of Rh-123 efflux (increase in accumulation), exhibition of a synergistic interaction by all compounds with epirubicin and enhancement of the antiproliferative effect of epirubicin	Duarte et al. (2008)
	Totarol	*Staphylococcus aureus* SA-K3092 (Totarol-resistant mutant overexpressing norA)	Checkerboard combination studies using ethidium bromide (EtBr) and totarol, EtBr efflux assay, modulatory activity of totarol at half the MIC	Reduction of the MICs of selected antibiotics by subinhibitory concentrations (suggesting that it may be an efflux pump inhibitor), reduction of ethidium efflux and ethidium MIC in SA-K3092	Smith et al. (2007)
	Tuckeyanol A, B	L5178/*MDR1*	Rh-123 accumulation assay, in vitro antiproliferative effect in combination with epirubicin using checkerboard microplate method	Inhibition of Rh-123 efflux (increase in accumulation), exhibition of a synergistic interaction by both compounds with epirubicin and enhancement of the antiproliferative effect of epirubicin	Duarte et al. (2008)
	Welwitschene	L5178Y‐MDR	MTT assay; Rh-123 accumulation assay	Increase in intracellular accumulation of Rh-123	Reis et al. (2016)
Triterpenoids, Saponins*	β-Amyrin	*MDR1*-transfected mouse lymphoma cells	Accumulation of Rh-123, accumulation of ethidium bromide (semi-automated ethidium bromide fluorometric method)	Increase in the accumulation of ethidium bromide (inhibitory activity against the P-gp transporter)	Martins et al. (2010)
	Cumingianol A, B, D	KB-C2	Cytotoxicity assay (MTT)	Enhancement of cytotoxicity against KB-C2 cells in the presence of colchicine	Kurimoto et al. (2011a)
	Cumingianol D	MCF7	Cytotoxicity assay (MTT)	Moderate cytotoxicity	Kurimoto et al. (2011a)
	Deacetylnomilin	CEM/ADR5000, Caco-2	Measurement of DOX cytotoxicity (reversal assay), Rh-123 efflux assay	Inhibition of Rh-123 efflux in CEM/ADR5000 cells, increase in DOX cytotoxicity in Caco-2 cells	El-Readi et al. (2010)
	Dyscusin A	KB-C2	Cytotoxicity assay (MTT)	Enhancement of cytotoxicity against KB-C2 cells in the presence of colchicine	Kurimoto et al. (2011b)
	Glycyrrhetinic acid (Enoxolone)	KB-C2, KB/MRP	DNR and calcein accumulation	Increasing DNR (inhibits the P-gp-mediated efflux of DNR) and calcein (inhibits the MRP1-mediated efflux of calcein) accumulation	Nabekura et al. (2008a)
	Glycyrrhizin*	Rats jejunal membrane in vitro	P-gp stimulation/inhibition profiles using a P-gp-dependent ATPase assay	Biphasic effect (stimulation at low concentration and inhibition at high concentration)	Najar et al. (2010)
	Limonin	CEM/ADR5000, Caco-2	Measurement of DOX cytotoxicity (reversal assay), Rh-123 efflux assay	Inhibition of Rh-123 efflux in CEM/ADR5000 cells, enhancement of DOX cytotoxicity in CEM/ADR5000 and Caco-2 cells	El-Readi et al. (2010)
	Obacunone	MES-SA/DX5, HCT15	Cytotoxicity assay in the presence of PTX	Significant inhibition of the P-gp MDR activity	Min et al. (2007)
	Oleanolic acid	Rats jejunal membrane in vitro	P-gp stimulation/inhibition profiles using a P-gp-dependent ATPase assay	Stimulatory effect on P-gp ATPase activity	Najar et al. (2010)
		*MDR1*-transfected mouse lymphoma cells	Accumulation of Rh-123, accumulation of ethidium bromide (semi-automated ethidium bromide fluorometric method)	Increase in the accumulation of ethidium bromide (inhibitory activity against the P-gp transporter)	Martins et al. (2010)
	Sinocalycanchinensin E	KB-C2	Cytotoxicity assay (MTT)	Enhancement of the cytotoxicity against KB-C2 cells in the presence of colchicine	Kashiwada et al. (2011)
	Ursolic Acid	KB-C2, KB/MRP	Accumulation assay of DNR and Rh-123 in KB-C2 and calcein in KB/MRP cells, ATPase activity assay, resistance to VBL cytotoxicity by a calorimetric assay	Increase in the accumulation of DNR and Rh-123 in KB-C2 cells, stimulation of P-gp ATPase activity	Nabekura et al. (2010)
	Uvaol	*MDR1*-transfected mouse lymphoma cells	Accumulation of Rh-123, accumulation of ethidium bromide (semi-automated ethidium bromide fluorometric method), checker-board assay (for the study of synergism between uvaol and DOX)	Increase in the accumulation of Rh-123 and ethidium bromide (inhibitory activity against the P-gp transporter), synergism with DOX cytotoxicity	Martins et al. (2010)
Steroids, Saponins*	Alisol B 23-acetate	HepG2-DR, K562-DR	Cellular Rh-123 and DOX accumulation, photoaffinity labeling of P-gp with [^125^I]iodoarylazidoprazosin, P-gp ATPase activity,	Enhancement of VBL toxicity, restoration of the activity of VBL in causing G2/M arrest, increase in DOX accumulation, delay of Rh-123 efflux, inhibition of the photoaffinity labeling of P-gp by [^125^I]IAAP, stimulation of the P-gp ATPase activity	Wang et al. (2004)
	11α-O-benzoyl-12β-O-acetyltenacigenin B	HepG2/DOX	MDR reversing potential evaluation (comparing IC_50_ values of an anticancer drug in the absence or presence of 11-Alpha-O-benzoyl-12β-O-acetyl-tenacigenin B)	Increase in the sensitivity of HepG2/Dox cells to the antitumor drugs DOX, VBL, puromycin, and PTX	Hu et al. (2008)
	DTN	Caco-2, CEM/ADR5000	MTT assay using, Rh-123 accumulation assay using fluorospectroscopy	Reversal of DOX resistance, reduction of the IC_50_ value of DOX	Eid et al. (2013)
	Ginsenoside M1, M4 and M12 (hydrolyzed metabolites of ginsenoside)	KB-C2	DNR accumulation, verapamil-induced ATPase activity (for M4)	Increase in DNR accumulation by M1, M4 and M12; decrease of ATPase activity by M4 (most potent substance in this study)	Kitagawa et al. (2007)
	Ginsenoside Rc and Rd	Multidrug resistant mouse lymphoma cells	–	Moderate reduction of the activity of the efflux pump	Berek et al. (2001)
	Ginsenoside Rg_3_*	KBV20C	Rh-123 retention assay, photo-affinity labeling with [^3^H]azidopine, [^3^H]VBL accumulation, cytotoxicity assay using Sulforhodamine B cell staining method	Increase in accumulation of Rh-123, inhibition of [^3^H]VBL efflux, prevention of binding of [^3^H]azidopine to P-gp, restoration of the sensitivity of KBV20C cells to DOX, COL, VCR, and VP-16	Kim et al. (2003)
	Ginsenosides Rg1, Rc, Rd, Re*	Multidrug resistant mouse lymphoma cells	–	Moderate inhibitory effect on the drug efflux pump	Molnár et al. (2000)
	Guggulsterone	KB-C2, KB/MRP	Accumulation assay of DNR and Rh-123 in KB-C2 cells and calcein in KB/MRP cells, ATPase activity of P-gp and MRP1	Increase in Rh-123 and DNR accumulation in KB-C2 cells, inhibition of Rh-123 efflux from KB-C2 cells, increase in the accumulation of calcein in KB/MRP cells, stimulation of ATPase activities of P-gp and MRP1	Nabekura et al. (2008c)
	Methylprototribestin*	L5178/*MDR1*	Rh-123 accumulation, checkerboard microplate method to study the interaction (MDR reversal effect) between the methylprototribestin and DOX	Increase in Rh-123 accumulation, synergistic interaction between methylprototribestin and DOX	Ivanova et al. (2009)
	Protopanaxatriol ginsenosides*	AML-2/D100	Accumulation assay of DNR, [^3^H]-azidopine photolabeling of P-gp	Reversing the resistance to DNR, increase in DNR accumulation, inhibition of [^3^H]-azidopine photolabeling of P-gp	Choi et al. (2003)
	β-sitosterol-O-glucoside*	CEM/ADR5000, Caco-2	Measurement of DOX cytotoxicity (reversal assay), Rh-123 efflux assay	Inhibition of Rh-123 efflux in CEM/ADR5000 cells, increase in the cytotoxicity of DOX in Caco-2 cells	El-Readi et al. (2010)
		Caco-2, CEM/ADR5000	MTT assay, Rh-123 accumulation assay using fluorospectroscopy	Reversal of DOX resistance in both cell lines, synergism	Eid et al. (2013)
		Caco-2, CEM/ADR5000	MTT assay, Rh-123 accumulation assay using fluorospectroscopy	Sensitization of cell lines, enhancement of cytotoxicity, light reduction of the IC_50_ value of DOX, synergism in the Caco-2 cells	Eid et al. (2013)
	Stigmasterol	Caco-2, CEM/ADR5000	Measurement of DOX cytotoxicity (reversal assay), Rh-123 efflux assay	Inhibition of Rh-123 efflux in CEM/ADR5000 cells, increase in the cytotoxicity of DOX in Caco-2 cells	El-Readi et al. (2010)
	Tenacigenin B: P8, P26 and P27	SW620/Ad300 (P-gp-overexpressing) MCF-7/VP (MRP-1-overexpressing) MCF-7/FLV1000 (ABCG2-overexpressing)	MDR reversal effect on cytotoxicity of anticancer drugs DOX (substrate of P-gp and MRP1) and MTX (substrate of ABCG2), flow cytometry-based efflux assay of Rh-123 (P-gp substrate), calcein AM (MRP1 substrate) and PhA (ABCG2 substrate)	Effective in circumventing MDR mediated by P-gp, MRP1 and ABCG2, inhibition of P-gp efflux activity, increase in intracellular concentration of the substrate drugs through the inhibition of MRP1- and ABCG2-mediated efflux	To et al. (2017)
	Tenacigenin B: P2, P3 and P6	SW620/Ad300 (P-gp-overexpressing), MCF-7/VP (MRP-1-overexpressing), MCF-7/FLV1000 (ABCG2-overexpressing)	MDR reversal effect on cytotoxicity of anticancer drugs DOX (substrate of P-gp and MRP1) and MTX (substrate of ABCG2), flow cytometry-based efflux assay of Rh-123 (P-gp substrate), calcein AM (MRP1 substrate) and PhA (ABCG2 substrate)	Effective in circumventing MDR mediated by P-gp and MRP1, inhibition of P-gp efflux activity by P2 and P6, increase in the intracellular concentration of the substrate drug via inhibition of MRP1-mediated efflux	To et al. (2017)
	Tenacigenin B: P1, P4, P5, P9 and P28	SW620/Ad300 (P-gp-overexpressing) MCF-7/VP (MRP-1-overexpressing) MCF-7/FLV1000 (ABCG2-overexpressing)	MDR reversal effect on cytotoxicity of anticancer drugs DOX (substrate of P-gp and MRP1) and MTX (substrate of ABCG2), flow cytometry-based efflux assay of Rh-123 (P-gp substrate), calcein AM (MRP1 substrate) and PhA (ABCG2 substrate)	Effective in circumventing P-gp-mediated MDR, P1 and P5 inhibition of P-gp efflux activity	To et al. (2017)
	Tenacissimoside A*	HepG2/DOX	DOX accumulation, Rh-123 and Hoechst 33342 efflux assay, cell cycle analysis, MDR reversing potential evaluation (comparing IC_50_ values of an anticancer drug in the absence or presence of Tenacissimoside A)	Increase in the sensitivity of HepG2/Dox cells to the antitumor drugs DOX, VBL, puromycin, and PTX, increase in DOX accumulation, enhancement of the action of DOX in causing G2/M arrest, inhibition of the efflux of Rh-123 and Hoechst 33342	Hu et al. (2008)
Tetraterpenes	Aurochrome	L5178/*MDR1*	Rh-123 accumulation assay	Moderate inhibition of P-gp	Gyémánt et al. (2006)
	Canthaxanthin	Caco-2, CEM/ADR5000	MTT assay, activity determination using Rh-123- and Calcein-AM retention assay	Significant enhancement of cytotoxicity and synergism using the following drugs: PTX, Cycloheximide, DOX, VBL, amphotericin-B, 5-FU, etoposide and cisplatine	Eid et al. (2012)
	Capsanthin	*MDR1* gene-transfected mouse lymphoma (L1210) cells	Rh-123 accumulation assay	Inhibition of the efflux, very active in MDR reversal effect	Molnár et al. (2006)
	Capsorubin	Human *MDR1*-gene-transfected mouse lymphoma (L1210) cells	Rh-123 accumulation assay	MDR reversal activity, enhancement of Rh-123 accumulation	Molnár et al. (2006)
	β-carotene	Caco-2, CEM/ADR5000	MTT assay, activity determination using Rh-123- and Calcein-AM retention assay	Synergism, significant enhancement of cytotoxicity of DOX, VBL, amphotericin-B, 5-FU, etoposide and cisplatine; substrate	Eid et al. (2012)
		Caco-2, CEM/ADR5000	MTT assay using, Rh-123 accumulation assay using fluorospectroscopy	Sensitization of cell lines, enhancement of cytotoxicity, strong reduction of the IC_50_ value of DOX and consequently increase of efficacy, synergism	Eid et al. (2013)
		Caco-2, CEM/ADR5000	MTT assay using, Rh-123 accumulation assay using fluorospectroscopy	Reversal of DOX resistance in both cell lines, synergism	Eid et al. (2013)
		*ABCB1*/Flp-In^TM^-293	MTT assay; calcein-AM accumulation assay	Increase in cytotoxicity of DOX; increase in intracellular concentration of calcein	Teng et al. (2016)
	Crocin	Caco-2, CEM/ADR5000	MTT assay, activity determination using Rh-123- and Calcein-AM retention assay	Synergism, enhancement of cytotoxicity of DOX, VBL, cisplatine; substrate	Eid et al. (2012)
	Diepoxycarotene	L5178/*MDR1*	Rh-123 accumulation assay	Moderate inhibition of P-gp	Gyémánt et al. (2006)
	Fucoxanthin	Caco-2, CEM/ADR5000	MTT assay, activity determination using Rh-123- and Calcein-AM retention assay	Significant enhancement of cytotoxicity and synergism using the following drugs: PTX, Cycloheximide, DOX, VBL, amphotericin-B, 5-FU, etoposide and cisplatine	Eid et al. (2012)
	Mutatochrome	L5178/*MDR1*	Rh-123 accumulation assay	Moderate inhibition of P-gp	Gyémánt et al. (2006)
	Retinoic acid	Caco-2, CEM/ADR5000	MTT assay, activity determination using Rh-123- and Calcein-AM retention assay	Significant enhancement of cytotoxicity and synergism using the following drugs: DOX, VBL, 5-FU, etoposide and cisplatine	Eid et al. (2012)
**Benzopyrones**					
Coumarins/coumaric acids	Auraptene	KB-C2 and KB/MRP	Accumulation assay of DNR in KB-C2 cells and calcein in KB/MRP cells, P-gp ATPase activity	Increase in the accumulation of DNR in KB-C2 cells, stimulation of the P-gp ATPase activity	Nabekura et al. (2008b)
		HT29	Cytotoxicity assay (MTT), real-time RT-PCR	Synergic effects with cisplatin, DOX and VCR, increase in the toxicity of applied radiations in auraptene pretreated cells, overexpression of p21 in auraptene pretreated cells after radiotherapy	Moussavi et al. (2017)
	Bergamottin	K562/ADM	[^3^H]vincristine uptake	Increase in [^3^H]vincristine uptake, weak MDR reversal activity	Ikegawa et al. (2000)
	Clausarin	K562/R7	DNR accumulation assay	Inhibition of P-gp-mediated drug efflux	Bayet et al. (2007)
	Dicynnamoyl-cis-khellactone	HepG2/Dox, K562/Dox	DOX accumulation and efflux assay, MDR reversing activity (cytotoxicity assay in the presence and absence of an anticancer drug), cell cycle distribution	Increase in DOX uptake and reduction of DOX efflux in HepG2/Dox cells, increase in cytotoxicity of anticancer drugs VBL, DOX, puromycin and PTX in HepG2/Dox and K562/Dox cells, enhancement of DOX-induced G2/M arrest in HepG2/Dox cells	Shen et al. (2006)
	Dihydroxybergamottin	K562/ADM	[^3^H]vincristine uptake	Increase in [^3^H]vincristine uptake, weak MDR reversal activity	Ikegawa et al. (2000)
	Phyllodulcin	KB-C2	DNR accumulation	Increase in DNR accumulation (inhibition of P-gp-mediated efflux of DNR)	Nabekura et al. (2008a)
	Praeruptorin A	HepG2/Dox, K562/Dox	MDR reversing activity (cytotoxicity assay in the presence and absence of an anticancer drug)	Increase in cytotoxicity of anticancer drugs VBL, DOX, puromycin and PTX in HepG2/Dox and K562/Dox cells	Shen et al. (2006)
**Others**					
Dibenzopyran	Magniferin	Rats jejunal membrane in vitro	P-gp stimulation/inhibition profiles using a P-gp-dependent ATPase assay	Biphasic effect (stimulation at low concentration and inhibition at high concentration)	Najar et al. (2010)
Phenols	[6]-Gingerol	KB-C2	Determination of DNR and Rh-123 accumulation	Increase in accumulation of DNR and Rh-123	Nabekura et al. (2005)
Glucoside	Parishin C	Lymphoma cells	–	Moderate reduction of the activity of the efflux pump	Berek et al. (2001)
Phenyl propanoid	Acteoside (Verbascosine)	Rats jejunal membrane in vitro	P-gp stimulation/inhibition profiles using a P-gp-dependent ATPase assay	Inhibitory effect on P-gp ATPase activity	Najar et al. (2010)

*Saponins are marked with asterisks.

Research on olomoucine, purvalanol and roscovitine revealed amongst other things the inhibitive potential of purine alkaloids on P-gp in the form of MDCKII-ABCB1 cells and human HCT-8 and HepG2 cells while olomoucine had the strongest effect of all (Cihalova et al., 2013). Several groups have run experiments with the benzazepine alkaloid capsaicin on different cell lines such as KB-C2, Caco-2 and CEM/ADR5000. The most relevant result was the inhibition of P-gp efflux in the presence of digoxin as substrate at non-toxic concentrations (Nabekura et al., 2005; Han et al., 2006; Li et al., 2018a).

Isoflavonoids and some flavonoids often act as phytoestrogens. Investigations revealed that hydroxyl groups at position C-5 and C-7 are an important property for the P-gp inhibitory effect of flavonoids, although hydrophobicity usually promotes the affinity (Sheu et al., 2010). Acacetin, genistein, kaempferol and naringenin, were examined by Imai et al. (2004) and it resulted in positive effects on K562 cells expressing BCRP. The cytotoxicity of MTX and 7-ethyl-10-hydroxycamptothecin was enhanced using phytoestrogens. In addition, genistein and naringenin enhanced the accumulation of topotecan in K562/BCRP cells. Also apigenin had strong reversal effect on BCRP-mediated MDR (Imai et al., 2004). In MCF-7 MX100 cells, which also overexpressed BCRP, apigenin had similar effect on the accumulation of the anticancer agent MTX (Zhang et al., 2004). However, naringenin, which differs from apigenin only in the saturation of C-2 and C-3, had a significant loss of potency in comparison to the latter due to the lack of a double bond (Conseil et al., 1998). Several groups experimented with the isoflavone and phytoestrogen biochanin A and its outcome on BCRP and P-gp in cell lines such as Panc-1, MCF-7 MX100, MDA435/LCC6, MCF-7/ADR, MDA435/LCCMDR1 and Caco-2. In all studies, an inhibition of drug efflux, an increase of accumulation and a potentiation of cytotoxicity were observed. A combination of biochanin A with some other flavonoids yielded additive effects (Nguyen et al., 2003; Zhang and Morris, 2003; Zhang et al., 2004; Sergent et al., 2005). Nobiletin and tangeretin prevented the P-gp mediated efflux of [^3^H]vincristine (Ikegawa et al., 2000). Among other flavones with methoxyls they were examined by Ohtani et al. (2007) for the uptake potential of [^3^H]vincristine as a P-gp substrate. The MDR-reversing effect increased with the number of methoxyl moieties but with the exception of both at C-3′ and C-5′ position on B-ring. In this case there was even a decrease of MDR-reversing potency. Chrysin is a hydroxyflavone with simple structure which has also been examined by a number of groups. The competitive inhibition of the MRP-2 pumps in Caco-2 cells and an antiproliferative effect of chrysin are certain (Gyémánt et al., 2005; Sergent et al., 2005). Moreover, a prevention of BCRP and P-gp mediated efflux took place in MCF-7 MX100 cells and L5178 cells, respectively (Zhang et al., 2004; Zhang et al., 2005; Gyémánt et al., 2005). A prenyl or geranyl group at C-6 and C-8 of chrysin, and a prenyl in both positions ensured an improvement of the binding affinity to P-gp. In addition, there was more potency for inhibition after geranylation than prenylation which could be due to its level of lipophilicity (Di Pietro et al., 2002). In contrast, glycosylation taking place at any position tested, dramatically decreased the binding affinity of flavonoids (Sheu et al., 2010). Another way to gain binding affinity toward the C-terminal nucleotide-binding domain of P-gp, were other kinds of alkylation of chrysin including methyl, benzyl, isopropyl and 3,3-dimethylallyl (Boumendjel et al., 2002).

Several experiments have been carried out for the flavan epigallocatechin gallate (EGCG), a derivative of epigallocatechin (EGC) abundant in green tea. EGCG has been tested on different cells such as liver cancer cells Bel-7404/DOX, the colon cancer cell line Caco-2, leukemia cells CEM/ADR5000, endocervical adenocarcinoma cells KB-C2 and others. An increase of drug accumulation and sensitization in these and several other cell lines was observed (Kitagawa et al., 2004; Liang et al., 2010; Eid et al., 2013; Reyes-Esparza et al., 2015). Particularly substitutions at D-ring had positive effect on EGC and derivatives. A derivative of EGC with three methoxy groups in cis-configuration at D-ring and an oxycarbonylvinyl as its connection to C-3 led to a higher potency. This structure only regulated P-gp and could not affect BCRP or MRP1 (Wong et al., 2015).

While curcumin is a promising agent for liver protection and inhibition of cancerous cell growth, other curcuminoids like demethoxycurcumin and tetrahydrocurcumin have being studied more closely. They inhibited the efflux of chemptherapeutics and consequently increased cellular accumulation of drugs (Limtrakul et al., 2007; Ampasavate et al., 2010).

**Figure 5:**
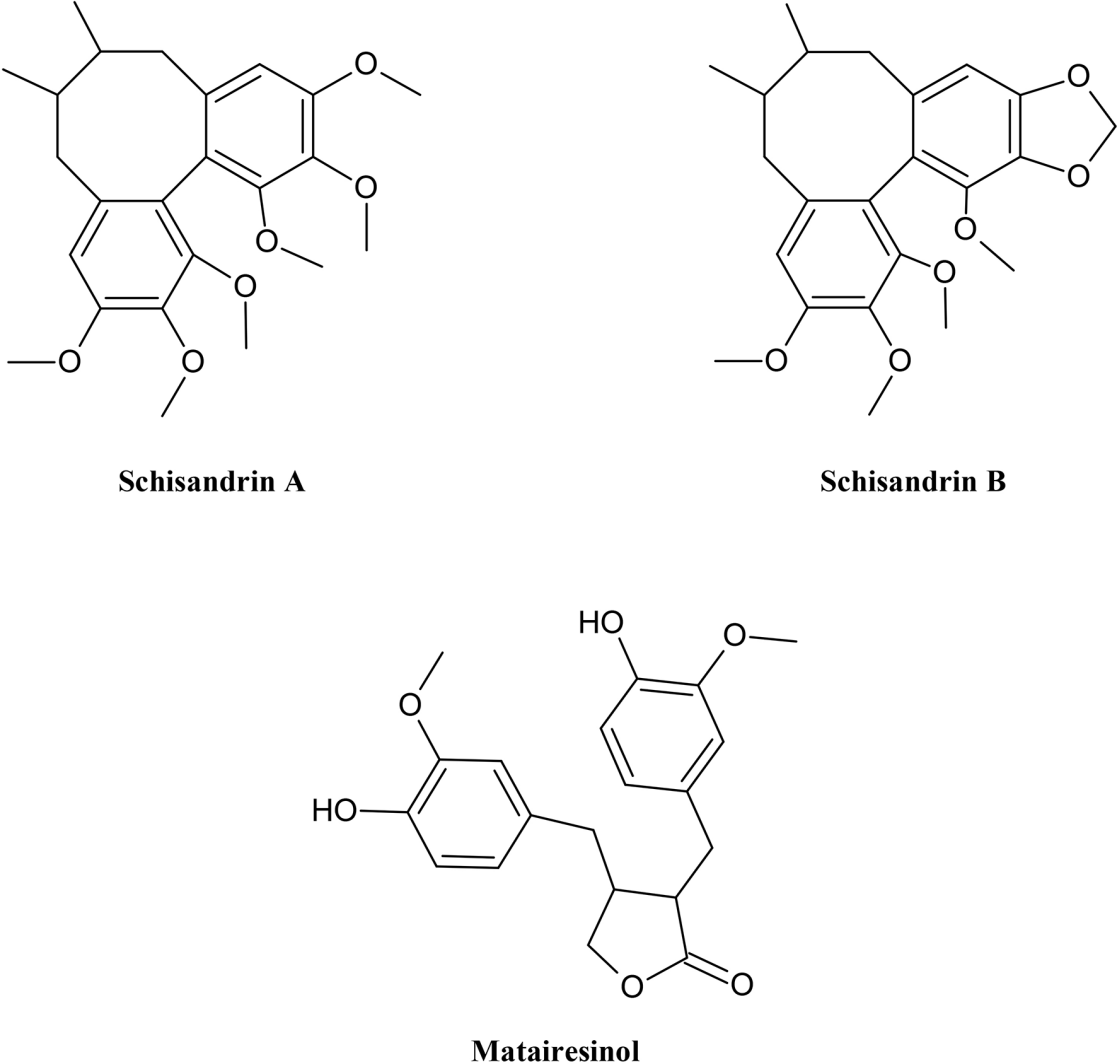
Chemical structures of some selected lignans with MDR reversal effects.

Among phenylpropanoids, some lignans (**Figure 5**) showed an inhibitory effect on P-gp. Schisandrin A was used on Caco-2 cells resulting in an increase in apical-to-basal transport and cytotoxicity Yoo et al. (2007b). A study which examined the structure activity of lignans showed the benefit of the absence of a hydroxyl group in position C-8 as in schisandrin A and γ-schizandrin for the function as p-gp inhibitor. In addition, a higher effect was seen in R-configurated biaryl than in the S-configuration (Slaninová et al., 2009). The reversal of cytotoxicity in CEM/ADR 5000 and Caco-2 cells was moreover successful by using menthol or thymol, monoterpenes obtained from volatile oils. They could reduce the IC_50_ value of doxorubicin which enhances the effectiveness of the drug (Eid et al., 2013).

Reis et al. (2016) reported that jatrophane diterpenes including esulatin M, epoxywelwitschene, welwitschene, and euphowelwitschine A were more efficative in MDR cells than the positive control verapamil. In comparison to the known MDR modifier verapamil, euphoboetirane C, D, E, F, and G extracted from *Euphorbia boetica* showed multifold P-gp modulatory activity in L5178Y‐MDR cells (Neto et al., 2019). Three types of latilagascenes (G, H and I), macrocyclic diterpene esters, were effective in L5178 cells expressing P-gp as transport inhibitors (Duarte et al., 2008). All successfully tested diterpenes mentioned above had a cyclopentane moiety in their structure. An enormous increase of activity was observed due to their saturation. Moreover, there was lower MDR-reversing activity mostly when the number of three hydroxyls was exceeded, especially in position 5, 7, 9 and 12. Nevertheless hydropxyls at C-1, C-13, C-14 and C-15 had not any negative effect on their function (Zhu et al., 2016). Vasas et al. (2011) observed that the lack of oxygenation at C-2 increased the MDR-reversing activity in diterpene jatrophanes such as 3β,5α,15β-triacetoxy-7β-isobutanoyloxyjatropha-6(17),11E-diene-9,14-dione and 3β,5α,15β-triacetoxy-7β-isobutanoyloxy-9α-nicotinoyloxyjatropha-6(17),11-dien-14-one. Furthermore, they stated that the presence of benzoyl or nicotinoyl group at C-2 instead of an acetyl group increases the potency of jatrophane diterpenes. The reversal of MDR was noticed in some triterpenoids including ursolic acid tested in KB-C2 cells (Nabekura et al., 2010), and deacteylnomilin and limonin in CEM/ADR5000 cells (El-Readi et al., 2010). A study on triterpenoids such as tormentic acid and derivatives suggests that an acetylation of C-2 of a terpenoid can cause an increase of activity, while this is not the case with the acetylation of C-3 (da Graça Rocha et al., 2007). Carotenoids, a group derived from tetraterpenes, are substrates for ABC transporters and can effectively modulate MDR in cancer cells (Eid et al., 2012). A few types such as β-carotene (Eid et al., 2013) or the xanthophyll member capsorubin showed MDR reversal activity (Molnár et al., 2006). To mention another important group with aromatic members, coumarins and coumaric acids are benzopyrones which we presented in **Tables 1** and **2**. Auraptene for example not only caused an enhancement of drug uptake (Nabekura et al., 2008b); its effect even was of a synergistic kind with drugs such as cisplatin and VCR (Moussavi et al., 2017). Kasaian et al. (2015) examined fifteen sesquiterpene coumarins. Considering the structure-activity relationship they described that ring-opened drimane-type sesquiterpene coumarins such as lehmferin, farnesiferol B and farnesiferol C showed the best P-gp inhibitory effects. Their study also revealed that farnesiferol C inhibited the Rh-123 efflux even more than the positive control verapamil.

**Table 2:** Phytochemicals Modulating Transporter or Protein Expression.

The effects of secondary metabolites on different cell lines expressing ABC-transporters – Regulation of the expression					
	Substance	Cell line	Assay system	Result	Reference
**Alkaloids**					
Quinolines, Isoquinolines, Quinazolines	Berbamine	K562/ADR	P-gp expression using flow cytometry, mdr-1 gene expression using RT-PCR	Reduction of *MDR1* gene expression	Dong et al. (2004)
	Berberine	OC2 and KB (oral-), SC-M1 and NUGC-3 (gastric-), COLO 205 (colon cancer cell line)	Pgp-170 protein expression using flow cytometry	Upregulation of P-gp expression in tested cell lines, decrease in retention of Rh-123	Lin et al. (1999a)
		Hep3B, HepG2, HA22T/VGH	Rh-123 retention and MDR1 transporter level using flow cytometry	Increase in MDR1 transporter level in HepG2, Hep3B, and HA22T/VGH cells, decrease in retention of Rh-123 in HepG2	Lin et al. (1999b)
		A10 (rat vascular smooth muscle cells)	*MDR1a* and *MDR1b* gene expression by RT-PCR	Increase in expression of *MDR1a* and *MDR1b* mRNA	Suzuki et al. (2010)
		3Y1, dRLh-84, B16	Rh-123 retention using flow cytometry, MDR1a and MDR1b protein expression using western blot	Increase in MDR1a and MDR1b protein level, decrease in intracellular Rh-123 concentration	Suzuki et al. (2010)
	Coptisine	A10	*MDR1a* and *MDR1b* gene expression by RT-PCR, MDR1a and MDR1b protein expression using western blot, Rh-123 retention using flow cytometry	Increase in MDR1a and MDR1b mRNA and protein level, decrease in intracellular Rh-123 content	Suzuki et al. (2010)
		3Y1, dRLh-84, B16	Rh-123 retention using flow cytometry, MDR1a and MDR1b protein expression using western blot	Increase in the level of MDR1a and MDR1b protein, decrease in intracellular Rh-123 concentration	Suzuki et al. (2010)
	Fangchinoline	Caco-2, CEM/ADR5000	MDR reversal assay (effect on DOX cytotoxicity), Rh-123 accumulation assay, western blot (P-gp expression level)	Increase in intracellular Rh-123 accumulation, decrease in P-gp expression, synergism in combination with DOX	Sun and Wink (2014)
	Glaucine	MCF-7/ADR	MDR reversing activity, ADR and MTX efflux assay, real-time RT-PCR, P-gp and MRP1 ATPase activity assay	Inhibition of P-gp and *MRP1*-mediated efflux, suppression of the expression of *MDR1* and *MRP1* genes, reversion of the resistance of MCF-7/ADR to ADR and MTX, increase in P-gp and MRP1 ATPase activities	Lei et al. (2013)
	O-(4-ethoxyl-butyl)-berbamine	MCF-7/ADR	MCF-7/ADR sensitivity to ADR (MTT assay), Rh-123 retention using flow cytometry, expression of mdr-1gene using RT-PCR	Sensitization to ADR, increase in the accumulation of Rh-123, reduction of *MDR1* gene expression	Cheng et al. (2006)
	Palmatine	A10	*MDR1a* and *MDR1b* gene expression by RT-PCR	Increase in the expression of *MDR1a* and *MDR1b* mRNA	Suzuki et al. (2010)
		3Y1, dRLh-84, B16	MDR1a and MDR1b protein expression using western blot, Rh-123 retention using flow cytometry	Increase in the level of MDR1a and MDR1b protein, increase in intracellular Rh-123 content	Suzuki et al. (2010)
	Tetrandrine	Caco-2, CEM/ADR5000	MDR reversal assay (effect on DOX cytotoxicity), Rh-123 accumulation assay, western blot (P-gp expression level)	Increase in intracellular Rh-123 accumulation, decrease in P-gp expression, synergism in combination with DOX	Sun and Wink (2014)
		K562 (DOX-treated vs. tetrandrine+DOX-treated group)	mRNA expression using RT-PCR, detecting P-gp expression as well as Rh-123 accumulation by FACS	Decrease in DOX-induced *MDR1* mRNA expression, inhibition of DOX-induced P-gp expression, increase in Rh-123 accumulation	Shen et al. (2010)
Quinolizidine alkaloids	Matrine	MCF-7/ADR	Western blot for labelling P-gp and MRP1 proteins, fluorospectrophotometry for ADR accumulation assay	Reduction of P-gp expression, increase in intracellular accumulation of ADR, decrease in cell growth	Zhou et al. (2017)
Indoles and β-carbolines	Antofine	A549-PA	P-gp expression using western blot, *MDR-1* mRNA expression using RT-PCR, Rh-123 accumulation by FACS	Reduction of P-gp and *MDR-1* mRNA expression, increase in intracellular Rh-123 content, synergism with PTX	Kim et al. (2012)
	Ephedrine	K562/A02	*MDR1* gene expression using semi-quantitative RT-PCR, P-gp expression using western blot	Decrease in *MDR1* mRNA and P-gp expression	Gao et al. (2008)
	Indole-3-carbinol	Hepatocytes of mouse treated with combination of indole-3-carbinol and VBL/VCR (*in vivo*)	P-gp expression using western blot, quantitative stereology using immunohistochemical staining with anti-P-gp antibody	Inhibition of VBL/VCR-induced P-gp expression	Arora and Shukla (2003)
	Staurosporine	MDR KB-V1	Cytotoxicity, P-gp expression using western blot, *MDR1* gene expression by northern blot	Cytotoxicity synergism with verapamil, sensitization of cells to VBL when co-treated with verapamil, decrease in P-gp and *MDR1* gene expression	Sampson et al. (1993)
	Vauqueline	K562/A02	*MDR1* gene expression using semi-quantitative RT-PCR, P-gp expression using western blot	Decrease in *MDR1* mRNA and P-gp expression	Gao et al. (2008)
Piperidines, Pyrazines, Diketopiperazines	Piperine	MCF-7/Dox	*ABCB1* and *ABCG2* mRNA expression by semi-quantitative RT-PCR; fluorescent dye efflux assay, cytotoxicity assay (MTT)	Decrease in *ABCB1* and *ABCG2* mRNA expression, increase in intracellular Rh-123 and MTX accumulation, reversal of resistance to DOX and MTX	Li et al. (2011)
		A-549/DDP	*ABCC1* mRNA expression by semi-quantitative RT-PCR; fluorescent dye efflux assay, cytotoxicity assay (MTT)	Decrease in *ABCC1* mRNA expression, increase in intracellular DOX accumulation, reversal of resistance to DOX	
	Tetramethylpyrazine	MCF-7/Dox	DOX retention by flow cytometry, P-gp expression using western blot, *MDR1* expression using RT-PCR	Increase in DOX retention, reversal of resistance to PTX, VCR and DOX; decrease in P-gp and *MDR1* mRNA expression	Zhang et al. (2012)
		Pumc-91/ADM, T24/DDP	Cell viability assay using Cell Counting Kit-8, qRT-PCR for *MRP1* mRNA, western blot and immunofluorescence assay for MRP1 protein expression	Decrease in MRP1 protein and mRNA expression, reversal of MDR (increase in cytotoxicity of ADR and DDP)	Wang et al. (2016)
		BEL-7402/ADM	ADR accumulation by flow cytometry and HPLC, gene expression using RT-PCR, protein expression by western blot	Decrease in *MDR1*, *MRP2*, *MRP3* and *MRP5* mRNA and proteins expression, increase in ADR intracellular accumulation, increase in ADR cytotoxicity (MDR reversal effect)	Wang et al. (2010)
Acridone alkaloids	Gravacridonetriol	L5178/*MDR1*	Rh-123 retention, MTT antiproliferative assay, *MDR1* expression by RT-PCR	Decrease in Rh-123 efflux, increase in DOX cytotoxicity (synergism), decrease in *MDR1* mRNA expression	Rethy et al. (2008)
	Gravacridonediol monomethyl ether	L5178/*MDR1*	Rh-123 retention, MTT antiproliferative assay, *MDR1* expression by RT-PCR	Decrease in Rh-123 efflux, increase in DOX cytotoxicity (synergism), decrease in *MDR1* mRNA expression	Rethy et al. (2008)
Nucleosides	Clitocine	R-HepG2, MES-SA/Dx5	DOX retention by flow cytometry, western blot for P-gp and qRT-PCR for *MDR1* expression	Decrease in *MDR1* mRNA and P-gp expression, reversal of MDR (enhancement of DOX cytotoxicity), increase in DOX accumulation	Sun et al. (2012)
	Sulfinosine	NCI-H460/R, U87-TxR	DOX retention by flow cytometry, western blot for P-gp and RT-PCR for *MDR1* mRNA expression	Decrease in *MDR1* mRNA and P-g expression, increase in DOX accumulation, decrease in resistance to DOX (enhancement of DOX cytotoxicity)	Dačević et al. (2013)
Further alkaloids	Capsaicin	Caco-2	[^3^H]-digoxin retention, western blot for P-gp and RT-PCR for *MDR1* mRNA expression	Increase in P-gp and *MDR1* mRNA expression, increase in [^3^H]-digoxin efflux	Han et al. (2006)
	Homoharringtonine	CEM/E1000	MTT assay	Decrease in cell viability, modulation of MRP1-mediated MDR	Efferth et al. (2002)
**Polyphenols**					
Flavonoids	Ampelopsin	K562/ADR	MTT assay, P-gp expression by PE-labeled antibody, ADR accumulation using flow cytometry	Decrease in P-gp expression, increase in ADR cytotoxicity and intracellular accumulation, synergism with ADR	Ye et al. (2009)
	Baicalein	LS174T, HepG2	MTT assay, *MDR1* expression using real-time PCR	Decrease in cell viability, increase in *MDR1* expression in LS174T	Li et al. (2010)
	Epicatechingallate	Bel-7404/DOX	MTT assay, Rh-123 retention by flow cytometry, intracellular DOX content using fluorospectrophotometry, semi-quantitative RT-PCR, P-gp expression by FACS using anti-P-gp monoclonal antibody	Increase in DOX and Rh-123 accumulation, and DOX cytotoxicity (synergism), decrease in *MDR1* and P-gp expression	Liang et al. (2010)
	Epigallocatechin gallate	Bel-7404/DOX	MTT assay, Rh-123 retention by flow cytometry, intracellular DOX content using fluorospectrophotometry, semi-quantitative RT-PCR, P-gp expression by FACS using anti-P-gp monoclonal antibody	Increase in DOX and Rh-123 accumulation, and DOX cytotoxicity (synergism),decrease in *MDR1* and P-gp expression	Liang et al. 2010)
		MCF-7Tam	MTT assay, RT-PCR, western blot for P-gp and BCRP protein expression, Rh-123 and MTX accumulation	Reduction of cell proliferation, decrease in P-gp and BCRP expression, increase in MTX accumulation	Farabegoli et al. (2010)
		MCF-7	Cytotoxicity using MTS assay, P-gp protein expression using western blot and immunofluorescence microscopy, Rh-123 accumulation	Increase in Rh-123 accumulation, decrease in P-gp protein expression, reduction of cell viability	Reyes-Esparza et al. (2015)
	Quercetin	HL-60/ADM, K562/ADM	MDR reversal by MTT assay, RT-PCR, flow cytometry	Sensitization to DNR, decrease in MRP1 gene and protein expression	Cai et al. (2004; 2005)
		BEL/5-FU	Cytotoxicity assay; Rh-123 and ADR accumulation using flow cytometry; ABCB1, ABCC1, ABCC2 mRNA and protein expression using real-time PCR and western blot	Increase in sensitivity to chemotherapeutic drugs 5-FU, MMC and ADR; increase in Rh-123 and ADR accumulation; decrease in ABCB1, ABCC1 and ABCC2 mRNAs and proteins expression	Chen et al. (2018)
Curcuminoids	Bisdemethoxycurcumin	KB-V1	RT-PCR, western blot	Decrease in *MDR1* and P-gp expression	Limtrakul et al. (2004)
	Curcumin	SKOV3(TR)	Cytotoxicity in combination with PTX, western blot	Enhancement of PTX cytotoxic activity, reduction of P-gp expression	Ganta and Amiji (2009)
		HCT-8/VCR	MTT assay, Rh-123 retention, *MDR1* and *survivin* genes expression by RT-PCR	Reversal of MDR, decrease in *MDR1* expression, increase in substrate accumulation	Lu et al. (2013)
**Neo-/lignans**	Honokiol	MCF-7/ADR	MTT assay, Rh-123 retention, qPCR, P-gp expression by FACS	Increase in Rh-123 retention, reduction of *MDR1* and P-gp expression, increase in ADR cytotoxicity	Xu et al. (2006)
Dibenzocyclo-octadienelignans	Schisandrin A (Deoxyschizandrin)	KBV200, MCF-7/Dox, Bel7402	MTT assay (cytotoxicity in combination with DOX, VCR and PTX), DOX and Rh-123 retention, RT-PCR, western blot for P-gp expression	Increase in Dox and Rh-123 retention, reduction of *MDR1* and P-gp expression, sensitization to cytotoxic drugs	Huang et al. (2008)
		COR-L23/R	MTT assay, DOX retention by flow cytometry	Elevation of DOX cytotoxicity, increase in DOX accumulation	Slaninová et al. (2009)
	Schisandrin B/γ-Schizandrin	COR-L23/R	MTT assay, DOX retention by flow cytometry	Elevation of DOX cytotoxicity, increase in DOX accumulation	Slaninová et al. (2009)
**Terpenoids**					
Sesquiterpenes	Artesunate	CEM/E1000, CEM/VLB100	MTT assay, DNR retention assay by flow cytometry	Decrease in cell viability, increase in DNR accumulation, modulation of MDR1- and MRP1-mediated MDR	Efferth et al. (2002)
	EGb761	HepG2	*MDR1* expression by real-time RT-PCR	Induction of *MDR1* expression	Li et al. (2009)
Diterpenes	Ginkgolide A and B	HepG2	*MDR1* expression by real-time RT-PCR	Induction of *MDR1* expression	Li et al. (2009)
	Triptolide	In vitro (KB-7D, KB-tax) In vivo (KB-7D-,KB-tax-bearing mouse)	In vitro growth inhibition assay, western blot for MDR1 and MRP1 protein expression, in vivo tumor weight evaluation	Inhibition of cell growth, decrease in MDR1 and MRP1 protein expression, inhibition of tumor growth, decrease in tumor weight when combined with 5-fluorouracil (synergism effect)	Chen et al. (2010)
		DU145/ADM	MTT assay, RT-PCR, western blot	Increase in ADR cytotoxicity, reduction of *MDR1* mRNA and protein expression	Guo et al. (2013)
**Benzopyrones**					
Coumarins/coumaric acids	Pyranocoumarins	KB-V1	Sulforhodamine B cytotoxicity assay, DOX retention by flow cytometry, *MDR1* mRNA Expression using RT-PCR, P-gp expression by western blot	Synergism with DOX, VCR, puromycin and PTX; increase in intracellular accumulation of DOX; decrease in P-gp and *MDR1* mRNA expression	Wu et al. (2003)
**Phenols**					
	6-Gingerol, 10-Gingerol	PC3R	Cytotoxicity assay, MRP-1 protein expression using western blot	Reduction of cell survival, decrease in MRP-1 protein expression	Liu et al. (2017)
	6-Shogaol, 10-Shogaol	PC3R	Cytotoxicity assay, MRP-1 protein expression using western blot	Reduction of cell survival, decrease in MRP-1 protein expression	Liu et al. (2017)

The studies introduced phytochemicals with completely different basic structures, each showing both strong and ineffective members in targeting ABC-transporters. The molecules mentioned were a selection chosen due to their effectiveness, especially in relation to the substances to which they were compared.

### Phytochemicals Modulating Transporter or Protein Expression

The following discussion focusses on the question how far phytochemicals affect the expression of ABC transporters or proteins in cancer cell lines. A list of relevant publications is documented in **Table 2**. Although many investigations addressed the inhibitory effect of secondary metabolites on transporter activity, many phytochemicals regulate the expression of corresponding genes, including alkaloids, polyphenols, lignans, terpenes and benzopyrones. Berbamine caused a downregulation of *Mdr-1* expression in K562/ADR cells (Dong et al., 2004), and so did its derivative O-(4-ethoxylbutyl)-berbamine (Cheng et al., 2006). Glaucine not only functioned as inhibitor of transporters but also reduced the expression of *MDR1* and *MRP1* (Lei et al., 2013) while the isoquinoline alkaloids berberine and coptisine, mediated the expression of P-gp (Lin et al., 1999a). Tetramethylpyrazine downregulated the expression of MDR1, MRP2, MRP3 and MRP5 in BEL-7402/ADM cells (Wang et al., 2010). It has also reduced the expression of MDR1 and MRP1 in MCF-7/Dox and Pumc-91/ADM cells, respectively (Zhang et al., 2012; Wang et al., 2016). *MDR1* mRNA and P-gp expression were reduced in response to clitocine and sulfinosine in several cell lines, such as R-HepG2, MES-SA/Dx5, NCI-H460/R and U87-TxR (Sun et al., 2012; Dačević et al., 2013). Among polyphenols, EGCG mediated a reduction of P-gp and BCRP expression resulting in drug accumulation (Farabegoli et al., 2010). Curcumin enhanced the cytotoxicity of PTX and ADR, respectively against SKOV3(TR) and K562/A02 cells by reducing the expression of P-gp in both cell lines (Chang et al., 2006; Ganta and Amiji, 2009). While some diterpenes and sesquiterpenes induced the transporter expression (Li et al., 2009), others like triptolide suppressed the expression of MDR1 and MRP1 proteins in KB-7D and KB-tax cells (Chen et al., 2010). Beside the positive effect of some coumarins on the activity of ABC transporters, pyranocoumarin was tested on KB-V1 cells and showed a reduction of P-gp protein and MDR1 expression (Wu et al., 2003).

Athough no clear relationship could be established between the structure of a modulator with thre expression of a transporter protein, these findings can be relevant for clinical applications.

### Synergistic Combinations of Chemotherapeutics With Phytochemicals

In addition to affecting the activity or expression of transporters, phytochemicals can also reverse MDR in cancer cells through synergism. Phytochemicals causing synergistic interactions with anticancer drugs are documented in **Tables 1** and **2**. The two-drug combination of harmine with DOX not only showed an enhancement of cytotoxicity but a synergistic effect. Glaucine potentiated DOX toxicity in Caco-2 and CEM/ADR5000 cells and reversed their resistance to anticancer drugs. Glaucine exerted synergistic interaction with DOX and even more in a three drug combination with DTN (Eid et al., 2013). Euphoboetirane C, D, F, G, H and I extracted from *Euphorbia boetica* showed strong synergistic interactions with DOX in L5178Y‐MDR cells (Neto et al., 2019). Nobiletin and antofine showed synergistic interactions with PTX (Kim et al., 2012; Ma et al., 2015). Jatrophane diterpenes including euphowelwitschine A, euphowelwitschine B, epoxywelwitschene, esulatin M isolated from *Euphorbia welwitschii* demonstrated synergism with DOX in L5178Y‐MDR cells (Reis et al., 2016). Fangchinoline, tetrandrine, and pyranocoumarins has been shown to synergistically increase the cytotoxicity of DOX (Wu et al., 2003; Sun and Wink, 2014). These synergistic effects are probably caused by interference of the phytochemicals with ABC transporters and additional molecular targets in cancer cells (Wink et al., 2012). These studies show that a combination of substances can have a great advantage over using single drugs against cancer cells by exploiting synergism.

### *In Silico* Analysis of Phytochemicals as MDR Reversing Agents

*In Silico* experiments are promising methods for making predictions about interactions of molecules to the drug-binding site of a target such as P-gp, BCRP and MRP1. Molecular docking studies can help finding the phytochemicals with best affinity for the drug-binding site which should be used as lead molecules for further studies. The nucleotide-binding domains (NBD) of these ABC-Transporters are hydrophilic protein parts (Jones and George, 2002). In addition to essential hydrogen bonds which play a crucial role in maintaining the stability and function of biomolecules, modulators with hydrophobic moieties often show affinties to NBDs.

The benzolisoquinoline alkaloid tetrandrine was confirmed to have inhibitory capability. Its binding affinity is close to verapamil and their docking positions are similar. Main part of tetrandrine is fixed in a lipophilic pocket formed by the amino acids Ala729, Ala987, Ileu306, Ileu340, Leu339, Leu65, Leu975, Met69, Met986, Phe303, Phe336, Phe343, Phe728, Phe732, Phe983, Tyr307, Tyr310. Moreover, the positively charged methylamine moiety of tetrandrine formed a cation-π interaction with Phe343 and an aryl ether got into a π-π interaction with Phe336 (Liao et al., 2019).

Regarding in silico investigations, another subtype of alkaloids, piperine was examined by Syed et al. (2017) revealing that hydrophobic interactions with P-gp took place in following positions: Leu339, Met69, Met986, Phe72, Phe336, Phe728, Phe983, Tyr953 and Val982. Moreover, an H-bond with Tyr307 was established. Two piperine analogs were designed which showed better hydrophobic interaction with most of the amino acids mentioned (Syed et al., 2017).

Pharmacophore modeling which was carried out for acridones indicated that three aromatic rings and two H–acceptors which are given in position C-9 bearing a carbonyl group and N-10 were conducive for stable docking. Including designed analogs of acridone the oxygen of a morpholine moiety at a phenyl ring built a water bridge with Ser309 of P-gp and a carboxamide at C-4 helped docking with Phe343. This analog showed hydrophobic interactions with Ala229, Ala302, Ala342, Ile218, Ile221, Ile299, Ile306, Leu225 with even more than two interactions, Leu339, Phe343, and Val345 of P-gp (Gade et al., 2018).

Badhan and Penny (2006) worked on the main characteristics of the pharmacophore of flavonoids by in silico modeling. Flavonoids tested get located within the C-terminal NBD of P-gp binding to amino acids of the ATP pocket such as Tyr1044. The hydroxyl moiety at C-5 of natural flavonoids can form a water bridge with Lys1076 while the hydroxyl group in position 3 and the carbonyl group get into hydrogen bonds within the NBD. Hydroxyl groups at position C-7 led to important hydrogen bonds with amino acids for several flavonoids such as EGC and chrysin (Wongrattanakamon et al., 2017). Ring B gets involved in π- π interaction with Tyr1044. If benzyl, geranyl or prenyl groups added at C-6 and several substitutes added at C-8 position, the docking capability was enhanced. Molecular docking analysis demonstrated that apigenin binds to the ATP-binding site of P-gp through a hydrogen bond with LYS408 and therefore, interferes with binding and cleavage of ATP which are vital for the function of ABC transporters (Saeed et al., 2015). Comparing flavonols to their corresponding flavones such as kaempferol to apigenin, better docking properties have been found in flavonols due to the number of hydrogen bonds (Badhan and Penny, 2006). A number of naringenin derivatives such as hydrazones and azines were produced by Ferreira et al. (2018) to achieve an improved MDR reversal in P-gp and BCRP. There was a high selectivity for most members of these two groups. It has been shown that the active compounds bind to hydrophobic residues such as transmembrane helix 4 and 5 in the center of an active pocket of BCRP similar to fumitremorgin C, a common inhibitor of BCRP. The hydrazone of these derivatives, a continuation of imine, is located in a pocket in π- π interaction with Phe515 and a dipole-dipole interaction with Met541. The B-ring is in another lipophilic bag made of Phe545, Pro574 and Val516. An improvement in the interaction between the transporter MRP1 and the carbohydrazide derivatives of flavonones due to the resulting flexibility was also observed (Ferreira et al., 2018).

### Conclusions and Future Perspectives

Due to the increase of cancer cases and restrictions in therapy owing to the degree of harmfulness, effectiveness and the associated high costs, the development of new substances with less side effects and higher efficacy is required. So far, many structures from the plant kingdom have been discovered and used, such as, diterpene derivatives as taxanes and vinca alkaloids are prime examples with regard to antitumor therapy. Therefore, numerous secondary metabolites which already had positive effect on cell lines should be taken into account for further investigations, which is why we have tried to list them extensively in our tables.

Many phytochemicals including alkaloids, flavonoids, curcuminoids, stilbenoids, terpenes, carotenoids, lignans, and polyketides were examined for their pharmacological activity against MDR. In most of the studies it was possible to differentiate between more effective and less effective molecules. As the next step these molecules must be further studied to determine molecular mechanisms and to identify the pharmacophores. Some valuable results have been provided by structure-activity relationship studies or in silico modeling as shown in this article. Subtances which were superior to others should be selected for further research, so possible lead molecules can be developed. A further step to increase efficacy could be the use of new drug delivery methods and controlled release as with nanotechnologies. As shown in this review, the combination of two and more modulators of differing structures and mode of actions together with chemotherapeutics is another interesting approach. The resulting additive, but above all synergistic effects can be of great importance. They would allow reducing the dose of chemotherapeutics resulting in less side effects and a higher compliance of patients.

Summin up, many phytochemicals in the group of alkaloids, flavonoids, curcuminoids, stilbenoids, terpenes, carotenoids, lignans, and polyketides have been investigated for MDR-reversing activity. Phytochemicals can be a promising source of adjuvant chemicals against cancer, not at least because of their generally low cytotoxicity in the human body. The adjuvant uses of MDR reversing phytochemicals in combination with anticancer drugs may improve the treatment of multidrug resistant cancer types. The present review summarized reports of several secondary metabolites that are capable of synergistically reversing MDR and inhibiting chemotherapy-resistant cancer cells by affecting transporter activity and the expression of ABC transporter genes. Synergism would allow reducing the dose of chemotherapeutics resulting in less side effects and a higher compliance of patients. The efficacy of phytochemicals needs to be confirmed clinically, but nevertheless they already can be considered as the fourth generation of ABC transporter modulators.

## Author Contributions

BT and IS performed the literature search and wrote the first draft of the manuscript. MW revised and edited the manuscript. All the authors approved the final version of the manuscript.

## Funding

Ruprecht-Karls-Universität Heidelberg provided financial support within Open-Access Publishing Program.

## Conflict of Interest

The authors declare that the research was conducted in the absence of any commercial or financial relationships that could be construed as a potential conflict of interest.

## Abbreviations

ADR, adriamycin; CPT, camptothecin; DOX, doxorubicin; DNR, daunorubicin; DTN, digitonin; EGC, epigallocatechin; EGCG, epigallocatechin gallate; MDR, multidrug resistance; MTX, mitoxantrone; P-gp, P-glycoprotein; PTX, paclitaxel; Rh-123, rhodamine 123; VBL, vinblastine; VCR, vincristine; BCRP, breast cancer resistance protein; MRP, multidrug resistance protein.
